# UNG-1 and APN-1 are the major enzymes to efficiently repair 5-hydroxymethyluracil DNA lesions in *C. elegans*

**DOI:** 10.1038/s41598-018-25124-1

**Published:** 2018-05-01

**Authors:** Arturo Papaluca, J. Richard Wagner, H. Uri Saragovi, Dindial Ramotar

**Affiliations:** 1 Maisonneuve-Rosemont Hospital, Research Center, Université de Montréal, Department of Medicine, 5415 Boul. de l′Assomption, Montréal Québec, H1T2M4 Canada; 2 Département de Médecine Nucléaire et Radiobiologie, Faculté de Médecine et des Sciences de la Santé, Université de Sherbrooke, 3001 12 Avenue Nord, Sherbrooke, Québec, J1H5N4 Canada; 3 Lady Davis Institute for Medical Research, Jewish General Hospital, McGill University, Department of Pharmacology and Therapeutics, McGill University, Department of Pharmacology and Therapeutics, 3755 Chemin de la Côte Sainte-Catherine, Québec, Montréal H3T1E2 Canada

## Abstract

In *Caenorhabditis elegans*, two DNA glycosylases, UNG-1 and NTH-1, and two AP endonucleases, APN-1 and EXO-3, have been characterized from the base-excision repair (BER) pathway that repairs oxidatively modified DNA bases. UNG-1 removes uracil, while NTH-1 can remove 5-hydroxymethyluracil (5-hmU), an oxidation product of thymine, as well as other lesions. Both APN-1 and EXO-3 can incise AP sites and remove 3′-blocking lesions at DNA single strand breaks, and only APN-1 possesses 3′- to 5′-exonulease and nucleotide incision repair activities. We used *C. elegans* mutants to study the role of the BER pathway in processing 5-hmU. We observe that *ung-1* mutants exhibited a decrease in brood size and lifespan, and an elevated level of germ cell apoptosis when challenged with 5-hmU. These phenotypes were exacerbated by RNAi downregulation of *apn-1* in the *ung-1* mutant. The *nth-1* or *exo-3* mutants displayed wild type phenotypes towards 5-hmU. We show that partially purified UNG-1 can act on 5-hmU lesion *in vitro*. We propose that UNG-1 removes 5-hmU incorporated into the genome and the resulting AP site is cleaved by APN-1 or EXO-3. In the absence of UNG-1, the 5-hmU is removed by NTH-1 creating a genotoxic 3′-blocking lesion that requires the action of APN-1.

## Introduction

Endogenous and exogenous reactive oxygen species (ROS), such as superoxide radical anions and hydrogen peroxide generate hydroxyl radicals that react with DNA to induce a variety of DNA damage^1^. Hydroxymethyluracil (5-hmU) is a common oxidative DNA lesion induced by ROS and due to active DNA repair, it is usually present at relatively low levels in mammalian cells^1,2^. This modified base and its glucosylated derivative (base J) is also formed by enzymatic reactions in bacteriophage and protozoa^3^. In *Caenorhabditis elegans*, the main source of 5-hmU is likely ROS induced oxidation of thymine, which displays normal base pairing with adenine (5-hmU•A). Another potential source of 5-hmU involves the enzymatic oxidation of 5-methylcytosine (5-mC), which generates 5-hydroxymethylcytosine (5-hmC), a reaction that may lead to deamination by an activation-induced deaminase creating a mismatch with guanine (5-hmU•G). Efficient removal of 5-hmU would restore normal DNA base pairing, otherwise in the case of 5-hmU•G leads to a transition mutation from 5-methylcytosine to thymine^2,4,5^. However, this latter pathway is unlikely to occur in *C. elegans* because they lack 5-methycytosine, ten eleven translocation (TET) family enzymes, and other BER enzymes associated with DNA methylation (see below).

At least, four DNA glycosylases belonging to the base-excision DNA repair (BER) pathway have been identified that remove 5-hmU from the genome and these include (i) the single-strand specific monofunctional uracil DNA glycosylase 1, SMUG1^6^, (ii) the bifunctional DNA glycosylase/AP lyase NTH1 that also removes a variety of oxidatively modified bases^7^, (iii) the thymine DNA glycosylase TDG^8^, and (iv) the methyl-CpG binding domain protein 4, MBD4 DNA glycosylase^9^. These DNA glycosylases cleave the *N*-glycosidic bond between the oxidatively modified base and the sugar moiety to produce a C1′ hydrolyzed abasic sugar. The abasic site created by these DNA glycosylases, except for NTH1 with an associated β-lyase activity (see below), is incised by an apurinic/apyrimidinic (AP) endonuclease creating a 3′-hydroxyl group and 5′-deoxyribose phosphate. The latter is removed by the lyase activity of DNA polymerase β, which simultaneously with its DNA polymerase activity inserts the correct nucleotide leaving a nick that is sealed by a DNA ligase in a set of reactions that constitutes the BER pathway^10–12^.

Of the multiple DNA glycosylases involved in removing 5-hmU in various organisms, only NTH-1 that shares 67.4% similarity with human NTH1 (see Figure S1A) has been conserved in *Caenorhabditis elegans*^13^. Besides NTH-1, *C. elegans* has retained one other DNA glycosylases, namely uracil DNA glycosylase UNG-1 that shares 58.2% similarity with human UNG1 (see Figure S1B)^14,15^. It seems enigmatic that this multicellular organism conserved only two DNA glycosylases, NTH-1 and UNG-1, while the unicellular organisms *Escherichia coli* and the budding yeast *Saccharomyces cerevisiae* conserved eight and five, respectively, and humans retained even more, eleven^12^. There might be a rationale for the evolutionary conservation of only NTH-1 and UNG-1 in *C. elegans*. This organism does not harbor homologs of the enzymes that program the methylation of cytosine, i.e., the DNA (cytosine-5-)-methyltransferases DNMT1 or DNMT3 to form 5-mC, as an epigenetic mark^16,17^. Furthermore, a search of the *C. elegans* genome database revealed that it lacks the Ten Eleven Translocation proteins TET1, 2 and 3 that are required to hydroxylate 5-mC to form 5-hmC and further oxidation products 5-formylcytosine and 5-carboxylcytosine in a pathway to regenerate the nonmethylated cytosine^16,17^. The lack of the TET1, 2 and 3 proteins would also prevent the conversion of thymine to 5-hmU to create the base pair 5-hmU•A in this organism. Moreover, *C. elegans* does not appear to harbor an AID/APOBEC deaminase to convert 5-hmC to 5-hmU. Since the MBD4 DNA glycosylase co-localizes to heterochromatin sites in a DNA methylation-dependent manner^9^, it would seem less important for *C. elegans* to conserve a homolog of MBD4 because its genome has no or undetectable 5-mC.

Likewise, it would seem unnecessary for *C. elegans* to also conserve the thymine DNA glycosylase TDG, which would be required to remove T•G mispair formed by deamination of 5-mC in the 5-mC•G base pair. Indeed, *C. elegans* lacks both MBD4 and TDG, raising the possibility that 5-hmU lesions generated as a consequence of thymine oxidation would be processed by either a SMUG1-like and or the NTH-1 activity in *C. elegans*. However, *C. elegans* also lacks in its genome a gene encoding a SMUG1-like DNA glycosylase. Altogether, *C. elegans* appears to lack a system to methylate, hydroxylate and demethylate cytosine in a process that would lead to 5-hmU formation, as well as lacking three DNA glycosylases, SMUG1, TDG and MBD4, that would ordinarily remove 5-hmU. Therefore, we anticipate that the task of removing 5-hmU lesions from the genome of *C. elegans* would be a function devoted strictly to the NTH-1 DNA glycosylase.

The *C. elegans* NTH-1 has been expressed and purified from an *E. coli* expression system and shown to efficiently remove oxidatively modified bases such as thymine glycol, 5-formyluracil and 5-hmU from oligonucleotide substrates^13^. NTH-1 acts as a bifunctional DNA glycosylase/AP-lyase, and following the removal of the modified base, the resulting AP site is cleaved by its AP-lyase activity via a β-elimination reaction to produce a single strand break terminated with a bulky 3′-α, β unsaturated aldehyde^7^. This 3′-blocking lesion must be removed by one of the two conserved AP endonucleases/3′-diesterases, APN-1 and EXO-3, to produce a 3′-hydroxyl group for DNA repair synthesis^18–20^. If the 3′-blocking lesions are not efficiently removed, they can also generate DNA and protein crosslinks that become more deleterious than simple abasic sites^21^.

In this study, we set out to investigate whether *C. elegans* mutants lacking enzymes of the BER pathway would be sensitive to exposure of the nucleoside form of 5-hmU. We report the surprising finding that *ung-1*, and not *nth-1*, mutants showed a number of phenotypes that are associated with a defect in DNA damage response when the animals were challenged with 5-hmU, suggesting that UNG-1 is the major DNA glycosylase involved in processing 5-hmU lesions. Consistent with this observation, partially purified UNG-1 was capable of removing 5-hmU from a deoxyoligonucleotide stem-loop substrate. We further show that APN-1-, but not EXO-3, -deficient mutant animals were sensitive to 5-hmU exposure and the effects were more dramatic in *ung-1*; *apn-1(RNAi)* knockdown mutants. We propose that UNG-1 has the ability to remove 5-hmU and channel the resulting AP site to be cleaved by APN-1 or EXO-3. In the absence of UNG-1, the 5-hmU lesion is processed by NTH-1, which creates the toxic 3′-bocking group that must be repaired by APN-1.

## Results

### *C. elegans* mutants deficient in both APN-1 and EXO-3 are hypersensitive to DOX-, MMS- and CDDP-induced DNA lesions

**Figure 1:**
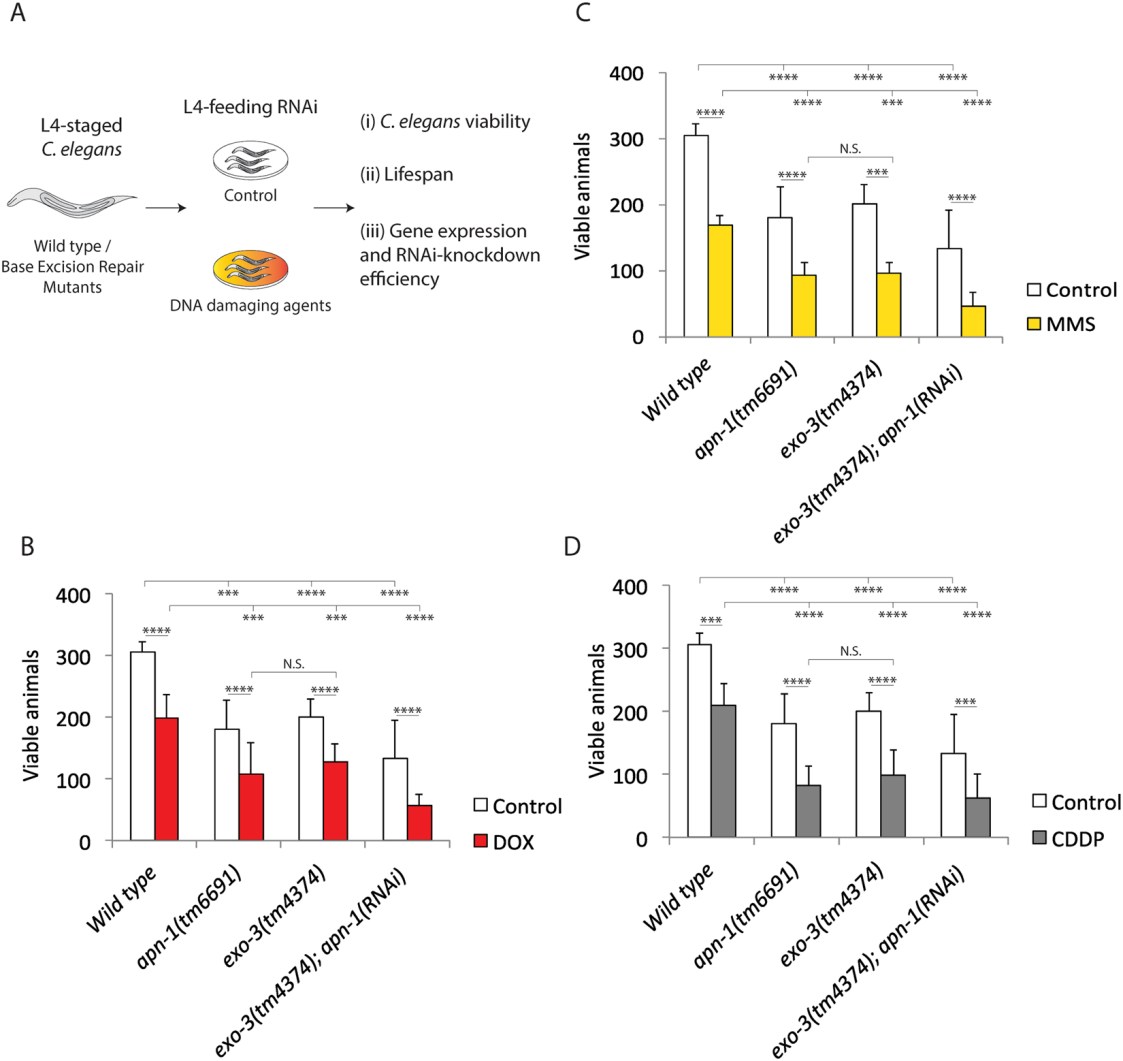
*C. elegans* mutants deficient in both APN-1 and EXO-3 are hypersensitive to DOX-, MMS- and CDDP-induced DNA lesions. (**A**) Scheme of the experimental design. (**B**,**C** and **D**), Brood size analyses of the indicated genotypes. The data are the mean ± S.D. of three independent experiments (*n* = 10 monitored for 3 days). Control; Wild type: 305 ± 17; *apn-1(tm6691)*: 181 ± 47; *exo-3(tm4374)*: 201 ± 29; *exo-3(tm4374); apn-1(RNAi)*: 134 ± 61. Exposed to drugs; (**B**) DOX 100 μM: Wild type: 199 ± 37; *apn-1(tm6691)*: 108 ± 50; *exo-3(tm4374)*: 128 ± 29; *exo-3(tm4374); apn1(RNAi)*: 57 ± 18. C, MMS 0.25 μM: Wild type: 168 ± 15; *apn-1(tm6691)*: 93 ± 20; *exo-3(tm4374)*: 97 ± 15; *exo-3(tm4374); apn1(RNAi)*: 46 ± 21. (**D**) CDDP 100 μM: Wild type: 210 ± 33; *apn-1(tm6691)*: 82 ± 31; *exo-3(tm4374)*: 98 ± 41; *exo-3(tm4374); apn1(RNAi)*: 63 ± 38. Error bars represent the S.D. Unpaired two-tail t-test ***P < 0.01; ****P < 0.0005 were considered to be statistically significant. N.S. = Non-Significant. DOX, doxorubicin; MMS, methyl methanesulfonate; and CDDP, cisplatin.

It has been shown that *C. elegans* mutants deleted for the *apn-1* gene are defective in the repair of damaged DNA that contains oxidative base lesions and AP sites^22^. These mutants exhibit elevated frequency of spontaneous mutations, as well as a short lifespan^22^. Since AP endonucleases serve as key components of the BER pathway, animals deleted for the *exo-3* gene also exhibit short lifespan^23^. These observations indicate that both enzymes bear the responsibility of repairing damaged DNA lesions to maintain *C. elegans* longevity. To date, no previous evidence exists indicating that the simultaneous deletion of the *apn-1* and *exo-3* genes would render the animals to even greater sensitivity to genotoxic agents due to the accumulation of unrepaired lesions in the genome in comparison to the single deletion mutants. We set out to investigate this idea by monitoring the viability of the animals by scoring the brood size and the lifespan following exposure to different DNA damaging agents (Fig. 1A). In this experiment, L1-staged wild type and mutant animals were systematically fed the HT115 bacteria harbouring the RNAi plasmid targeting *apn-1* and *exo-3* to score the brood size, and using the *apn-1* and *exo-3* gene deletion mutants *apn-1(tm6691)* and *exo-3(tm4374)* for comparison, respectively. As expected, the deletion mutants *apn-1(tm6691)* and *exo-3(tm4374)* exhibited a significant decrease in brood size in comparison to the wild type control animals (Fig. 1B–D, white bars) consistent with previous observations^22,23^. RNA-interference (RNAi)-driven depletion of *apn-1* in the *exo-3* deletion mutant *exo-3(tm4374)* caused the resulting *exo-3(tm4374); apn-1(RNAi)* knockdown mutant animals to exhibit nearly 65% decrease in brood size as compared to the single mutants *apn-1(tm6691)* and *exo-3(tm4374)* showing ~40% and 30% decreased in brood size, respectively (Fig. 1B–D, white bars). The effectiveness of the *apn-1(RNAi)* and *RNAi* against other genes (see below) was tested against the wild type (Figure S2A).

We investigated whether there would be a different requirement for APN-1 and EXO-3 molecular activities for processing DNA lesions produced by distinct DNA damaging agents. For this purpose, we exposed the animals to the DNA damaging agents doxorubicin (DOX), methyl methanesulfonate (MMS), and cisplatin (CDDP) that are known to create a range of DNA lesions that include oxidatively damaged bases, alkylated bases that are unstable, single and double strand breaks and interstrand crosslinks^24,25^. We used drug concentrations that allowed the animals to develop and found that DOX, MMS and CDDP induced nearly 50% decrease in brood size as observed for either the single deletion mutant *apn-1(tm6691)* or *exo-3(tm4374)* (Fig. 1B–D). The brood size decreased to 80% when the mutant *exo-3(tm4374)* knockdown for *apn-1(RNAi)* was exposed to the drugs (Fig. 1B–D). These results indicated that the repair of the lesions induced by the distinct DNA damaging agents were dependent upon the single and/or combined activity of APN-1 and EXO-3 as depletion of both enzymes resulted in an additive effect on the brood size. Thus, it would appear that these genotoxic agents are likely generating at least a common DNA lesion, such as indirect formation of AP sites from damaged base, which APN-1 and EXO-3 can compete to repair. In fact, it is known that oxidatively damaged bases are produced by doxorubicin and that the cytosine adjacent to CDDP-induced interstrand crosslinks can preferentially endure oxidative deamination to create uracil^24,25^.

### *C. elegans* apn-1, but not exo-3, mutants are sensitive to the nucleoside 5-hmU

**Figure 2:**
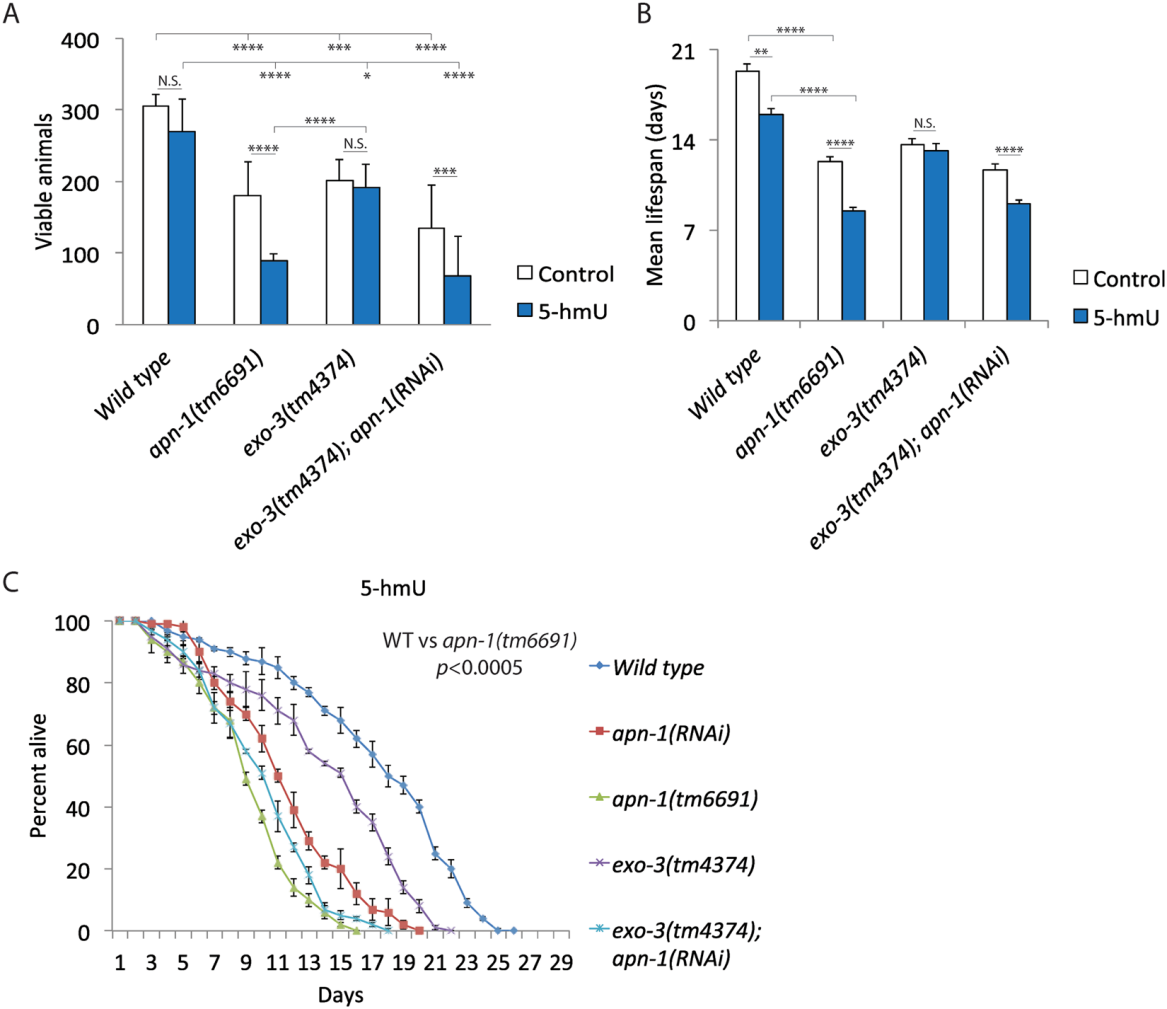
*C. elegans apn-1*, but not *exo-3*, mutants are sensitive to the nucleoside 5-hmU. (**A**) Brood size analysis of animals exposed to 5-hmU. Wild type: 269.8 ± 46; *apn-1(tm6691)*: 102 ± 21; *exo-3(tm4374)*: 171 ± 43; *exo-3(tm4374); apn1(RNAi)*: 68 ± 55. Control; Wild type: 305 ± 17; *apn-1(tm6691)*: 181 ± 47; *exo-3(tm4374)*: 201 ± 29; *exo-3(tm4374); apn1(RNAi)*: 134 ± 61. Error bars represent the S.D. Unpaired two-tail t-test ***P < 0.01; ****P < 0.0005 were considered to be statistically significant. N.S. = Non-Significant. (**B**) Mean lifespan of the indicated animals exposed to 5-hmU. (**C**) Kaplan-Meier survival plot showing the percentage of alive animals when the indicated genotypes were exposed to 5-hmU. L1-staged animals (*n* = 100) were exposed to 5-hmU (1 μM) and lifespan was blindly analyzed starting from young adult worms. The mean lifespan of two independent experiments is shown.

We have previously shown that APN-1, but not EXO-3, has the ability to process oxidized base DNA lesions, as well as exert a 3′ to 5′-exonuclease activity presumably to act on strand breaks with blocked 3′-ends^20,22^. Oxidation of thymine can lead to 5-hydroxymethyluracil (5-hmU), but as a mispair opposite adenine (5-hmU•A) ^2^. We assume that feeding *C. elegans* 5-hmU would lead to its conversion into the triphosphate form and subsequent incorporation into the genome as observed for mammalian cells^1^. Since the purified *C. elegans* NTH-1 enzyme has been shown to remove 5-hmU from oligonucleotide substrate followed by a β-elimination reaction to produce the product 3′-α, β unsaturated aldehyde, instead of an AP site^13^. We predict that this latter lesion would require the function of the 3′-diesterase of either APN-1 or EXO-3 or both for its removal. Following treatment with 5-hmU, we observed that the *apn-1(tm6691)* mutants exhibited nearly 50% decrease in brood size as oppose to *exo-3(tm4374)* that showed only 5% decrease in brood size (Fig. 2A).

However, when the *exo-3(tm4374); apn-1(RNAi)* knockdown mutant was exposed to 5-hmU, we observed the same deleterious effect caused by the *apn-1(tm6691)* mutation alone: close to 50% decrease in brood size (Fig. 2A), again suggesting that EXO-3 has no major role in processing the 5-hmU lesions. To further test this possibility, we examined whether the expression level of the *apn-1* and the *exo-3* genes would be affected by 5-hmU treatment. Interestingly, we observed a nearly 3-fold induction in the expression of the *apn-1* gene when the animals were exposed to 5-hmU, while the *exo-3* gene was unaffected (Figure S2B), consistent with the notion that APN-1, and not EXO-3, is the major AP endonuclease involved in processing the 5-hmU lesion.

We next checked whether exposure to 5-hmU would affect the longevity of the animals by measuring the lifespan of the wild type and the mutant worms in the absence and presence of the nucleoside. We noticed that the lifespan of the *apn-1(tm6691)* mutants was further decreased, but not that of the *exo-3(tm4374)* mutants, when exposed to 5-hmU as compared to the untreated (Fig. 2B,C vs. Figure S3A). Downregulation of *apn-1* by RNAi in the *exo-3(tm4374)* mutants yielded the *exo-3(tm4374); apn-1(RNAi)* knockdown mutants showing a shortened lifespan towards 5-hmU that was similar to the decreased lifespan observed for the *apn-1(tm6691)* single mutant (Fig. 2B,C). We interpret this observation to suggest that APN-1, and not EXO-3, is the predominant endonuclease that is recruited to process the damaged base 5-hmU when it is incorporated into the genome of *C. elegans*. Therefore, the diminished brood size and lifespan caused by 5-hmU in the *apn-1(tm6691)* mutants are attributed to unrepaired lesions in the mitotic and post-mitotic tissues, respectively.

### The apn-1(tm6691) mutant animals display increase spontaneous and 5-hmU-induced germ cell apoptosis

**Figure 3:**
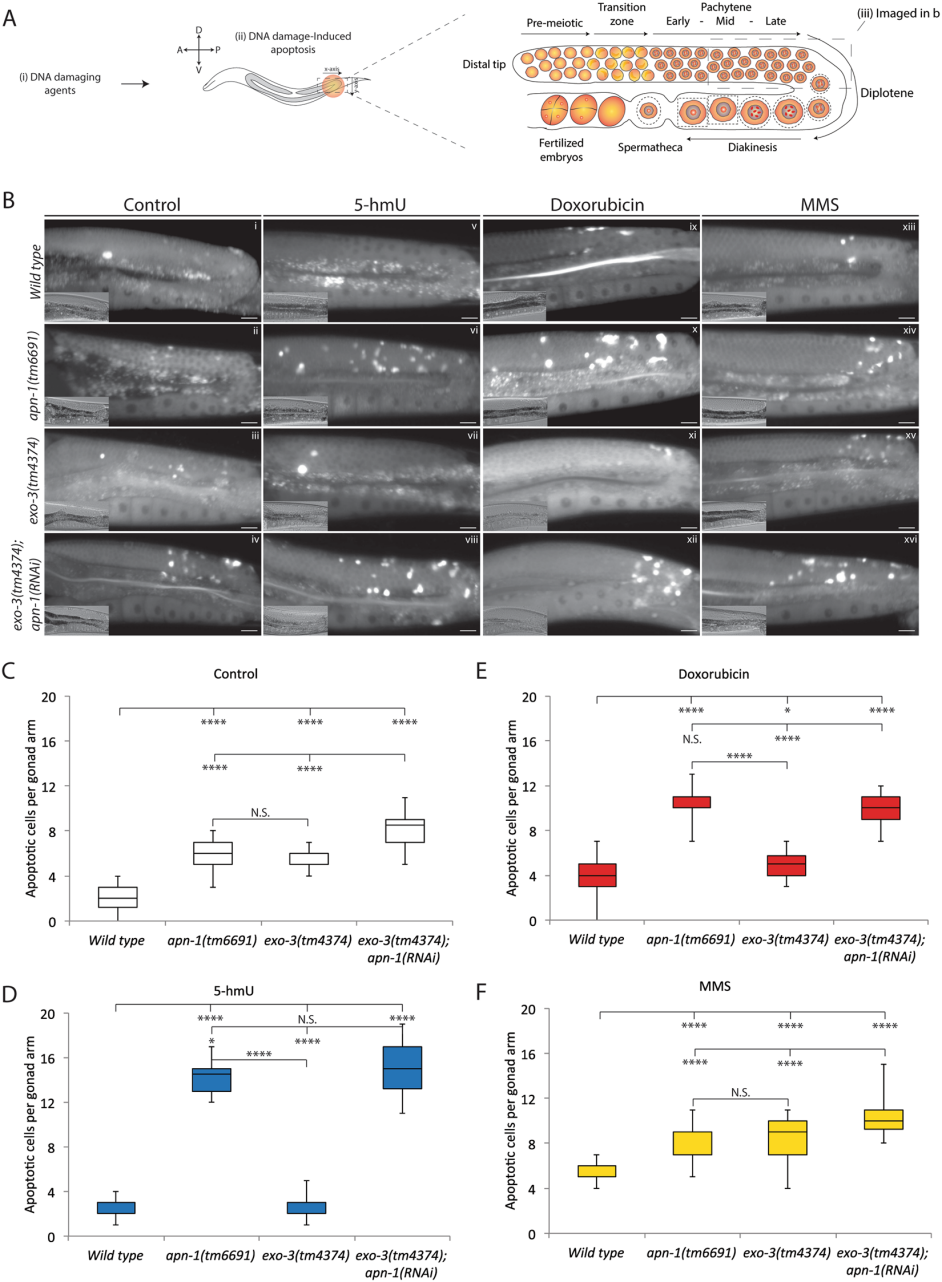
The *apn-1(tm6691)* mutant animals display increase spontaneous and 5-hmU-induced germ cell apoptosis. (**A**) Scheme showing the region of germ cells analyzed for apoptotic corpses in the posterior side of the gonad arm. (**B**) Representative images of acridine orange-stained and DIC (lower left) of control and drug-treated apoptotic corpses from the indicated genotypes. Apoptotic cell corpses were identified as bright spots correlating with raised-bottom-like refractive corpses shown on DIC images. Posterior is right and dorsal is top. Scale bar = 15 μm. (**C**–**F**) Box and whisker plots showing quantification of apoptotic corpses from control and drug-treated animal and displaying the maximum, minimum, upper & lower quartiles, and sample median. L4-stage animals were treated with (**D**) 5-hmU (1 μM), (**E**) DOX 100 μM and (**F**) MMS 0.25 μM, and apoptotic corpses were quantified the following day in the gonad arms of young adult staged worms. Statistical significance bars represent results of Mann-Whitney U-test of mean difference (*P < 0.05; **P < 0.01; ***P < 0.001 and ****P < 0.0001) computed from three independent experiments (*n = *30).

Like many other stem cell systems, *C. elegans* features a self-renewing germ cell population originated from a cellular lineage located at the distal tip (Fig. 3A)^26^. In these germ cells, differentiation occurs throughout distinct stages and they must maintain the integrity of the genome. When exposed to environmental insults, germ cells respond by using conserved DNA damage repair pathways that act to maintain genomic stability. Germ cells that are unable to repair damaged DNA undergo apoptosis and subsequent embryonic death^26–29^. We used this sensitive germ cell apoptosis assay to investigate whether 5-hmU would induce germ cell death in the animals and whether this effect would be enhanced in the absence of APN-1 and EXO-3. To do this, we utilized differential interference contrast (DIC) microscopy and DNA staining with acridine orange^30^ to quantify the levels of apoptosis in the proximal zone of the gonad arm *in vivo* (Fig. 3A). Consistent with previous reports^31^, we observed an average of 2.0 ± 1 apoptotic corpses per wild type animal (Fig. 3B[i]). In contrast, the *apn-1(tm6691)* and the *exo-3(tm4374)* mutants showed an average of 6.0 ± 1.3 and 5.5 ± 0.9 apoptotic corpses per animal, respectively (Fig. 3B[ii and iii] and C). Unlike the single mutants, the *exo-3(tm4374); apn-1(RNAi)* knockdown mutant depicted an average of 8.0 ± 1.5 apoptotic corpses per animal significantly higher than the wild type and the single mutants (Fig. 3B[iv] and C). The observation that the deficiency of both APN-1 and EXO-3 resulted in an additive effect on germ cells apoptosis, indicates that both of these enzymes function independently to promote base excision repair of spontaneous DNA damage in these germ cells.

On the basis of the above results, we challenged the wild type and mutant animals with 5-hmU, as well as the DNA damaging agents DOX and MMS that are known to induce germ cell apoptosis^22,28^. When the wild type animals were exposed to either 5-hmU, DOX and MMS they displayed an average 3.0 ± 1.0, 4.0 ± 2.0 and 5.0 ± 1.0 apoptotic cells per animal, respectively (Fig. 3B[v, ix and xiii] and D–F), suggesting that these doses are effective at causing slight genotoxicity to the germ cells of the wild type animals. In contrast, when the *apn-1(tm6691)* mutant animals were exposed to either 5-hmU, DOX and MMS they showed significantly higher levels of apoptotic cells as compared to both untreated and treated wild type animals (5-hmU: 14.3 ± 1.7; DOX: 10.4 ± 1.3; MMS: 8.5 ± 1.4) (Fig. 3B[vi, x and xiv vs. ii and i] and 3D–F). The high levels of apoptotic cells observed in the *apn-1(tm6691)* mutant exposed to 5-hmU is in agreement with previous studies showing that 5-hmU incorporates into the genome and triggers apoptosis in mammalian cells^32^. Thus, it would also appear that 5-hmU is incorporated into the genome of the germ cells and requires at least the DNA repair functions of APN-1.

Unlike the *apn-1(tm6691)* mutant, the *exo-3(tm4374)* mutants treated with either 5-hmU or DOX, respectively, showed an average of 2.7 ± 1.1 or 5.1 ± 1.2 apoptotic cells per animal, which was comparable to the wild type (Fig. 3B[vii and xi vs. v and ix] and D,E), suggesting that EXO-3 has minimal role compared to APN-1 in processing 5-hmU and DOX-induced DNA lesions. However, *exo-3(tm4374)* mutants exposed to MMS, depicted an average of 8.3 ± 1.8 apoptotic cells per animal similar to the *apn-1(tm6691)* mutants (8.5 ± 1.4) (Fig. 3B[xv vs. xiv] and F). In addition, the level of apoptotic cells was augmented when the *exo-3(tm4374); apn-1(RNAi)* knockdown mutant was exposed to MMS (10.5 ± 1.7) (Fig. 3B[xvi] and F). This observation indicates that EXO-3 can compete with APN-1 to repair MMS-induced DNA lesions, but not for the 5-hmU or the DOX-induced DNA lesions (see also for lifespan Figure S3). Collectively, our results suggest that (i) 5-hmU is incorporated into the genome of the germ cells and causes genotoxicity and (ii) APN-1 plays a key role in repairing 5-hmU lesions and not EXO-3.

### 5-hmU induces CED-1 engulfment of apoptotic germ cells

**Figure 4:**
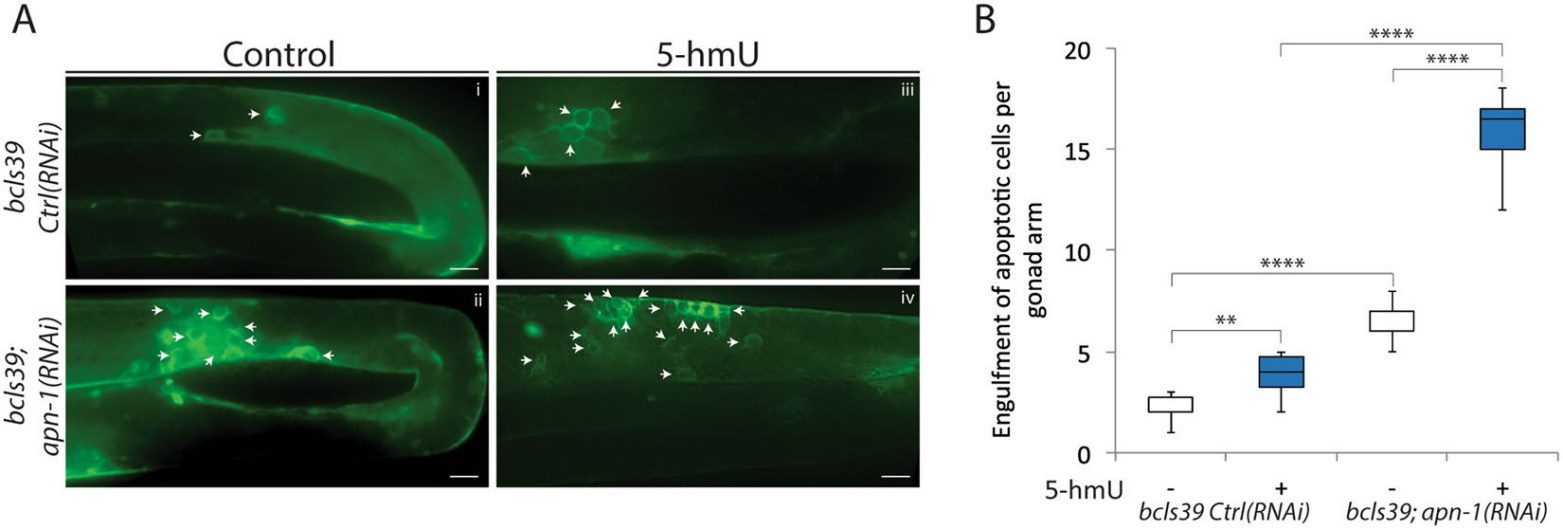
5-hmU induces CED-1 engulfment of apoptotic germ cells. (**A**) Representative images of control and 5-hmU-treated *bcls39* [CED-1::GFP] worms. The ring around the apoptotic corpses indicates the engulfment of apoptotic bodies shown by white arrows. Scale bar = 10 μm. (**B**) Box and whisker plots showing quantification of the engulfment of apoptotic bodies.

To ensure that the quantification of the germ cell death caused by 5-hmU is not a contribution from potential artifacts induced by the acridine orange staining method and or endogenous autofluorescence, we assessed the presence of apoptotic cells using a downstream component of the apoptotic pathway, CED-1. This protein engulfs apoptotic cells to signal phagocytic degradation^33^. As previously reported, we utilized an imaging method in which the *bcls39* strain carries the CED-1::GFP as a reporter of engulfed apoptotic cells^28,34^. This *bcls39* strain with control RNAi showed an average engulfment of 2.0 ± 1.0 apoptotic cells per animal (Fig. 4A[i]), whereas depletion of *apn-1* via RNAi in the *bcls39* reporter caused an increase average engulfment of 6.7 ± 0.9 apoptotic cells due to spontaneous DNA damage (Fig. 4A[ii]) and consistent with the acridine orange staining observed by the *apn-1(tm6691)* mutant (Fig. 3B[ii]). Furthermore, exposure of the *bcls39* reporter strain and the *bcls39; apn-1(RNAi)* to 5-hmU elevated the appearance of apoptotic cells (4.0 ± 0.8 and 16.0 ± 1.5 respectively) (Fig. 4A[iii and iv] and B), supporting the notion that indeed 5-hmU creates genotoxic lesions in the germ cells that must be processed by APN-1. To confirm that 5-hmU can promote apoptotic signaling, we tested several mutants deleted for key components of the apoptotic pathway including *cep-1*, *egl-1*, *ced-9*, *ced-4* and *ced-3*^26^. As expected, none of these mutants showed significant increase in apoptotic cells following exposure to 5-hmU (Figure S4A).

### ung-1, but not nth-1, mutants are sensitive to the genotoxic effects of 5-hmU

**Figure 5:**
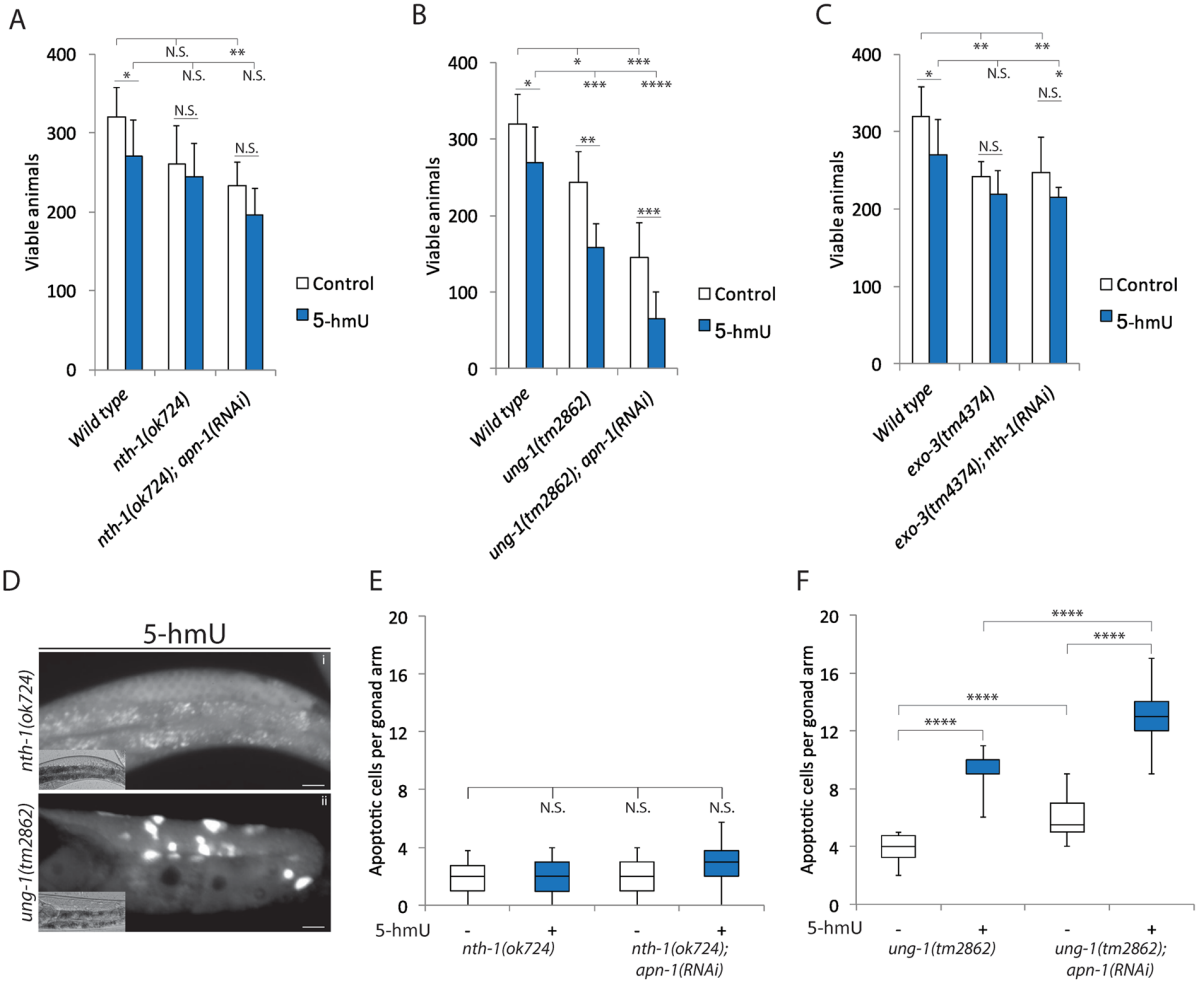
. *ung-1*, but not *nth-1*, mutants are sensitive to the genotoxic effects of 5-hmU. (**A**,**B** and **C**) Control and 5-hmU-treated animals for analysis of brood size as described in Fig. 1B–D. The data are the mean ± S.D. of three independent experiments (*n* = 10 monitored for 3 days). Error bars represent the S.D. Unpaired two-tail t-test ***P < 0.01; ****P < 0.0005 were considered to be statistically significant. N.S. = Non-Significant. (**A**) Control; Wild type: 319 ± 38; *nth-1(ok724)*: 260 ± 48; *nth-1(ok724); apn1(RNAi)*: 232 ± 30, and exposure to 5-hmU; Wild type: 269 ± 46; *nth-1(ok724)*: 244 ± 42; *nth-1(ok724); apn1(RNAi)*: 195 ± 33. (**B**) Control; Wild type: 319 ± 38; *ung-1(tm2862)*: 244 ± 39; *ung-1(tm2862); apn1(RNAi)*: 146 ± 45 and exposure to 5-hmU; Wild type: 269 ± 46; *ung-1(tm2862)*: 157 ± 32; *ung-1(tm2862); apn1(RNAi)*: 65 ± 35. (**C**) Control; Wild type: 319 ± 38; *exo-3(tm4374)*: 241 ± 19; *exo-3(tm4374); nth-1(RNAi)*: 247 ± 45 and exposure to 5-hmU; Wild type: 269 ± 46; *exo-3(tm4374)*: 219 ± 30; *exo-3(tm4374); nth-1(RNAi)*: 215 ± 12. (**D)**, Representative images of acridine orange-stained and DIC (lower left) of 5-hmU treated *nth-1(ok724)* and *ung-1(tm2862)* mutant animals as described in Fig. 2B. (**E**,**F**) Box and whisker plots showing quantification of apoptotic corpses from control and 5-hmU-treated of (**E**) *nth-1(ok724)* and (**F)** *ung-1(tm2862)* mutant animals and scored as in Fig. 2C.

So far only two DNA glycosylases UNG-1 and NTH-1 have been identified and partially characterized in *C. elegans*^13,15^. Both UNG-1 and NTH-1 function upstream of the APN-1 and EXO-3 AP endonucleases and catalyze the first step of the BER pathway. While purified UNG-1 removes uracil, purified NTH-1 acts on thymine glycol, 5-formyl uracil and 5-hmU^13,15^. We found that both *ung-1* and *nth-1* gene expression were elevated when wild type worms were treated with 5-hmU (2B). However, based on the biochemical activities, we anticipate that *nth-1* mutant would display sensitivity to 5-hmU. Survival analysis, scoring for brood size, revealed that the *nth-1(ok724)* mutant was not sensitive to 5-hmU exposure (Figure 5A) or showed increase in germ cell apoptosis under normal growth conditions or when treated with 5-hmU (2.0 ± 1.0 and 2.0 ± 1.0 respectively) (Fig 5D,E). In fact, these findings are consistent with a previous report showing that the *nth-1(ok724)* mutant do not show lifespan defects or sensitivities to oxidants such as hydrogen peroxide and paraquat^13^, raising the possibility that the burden of 5-hmU lesions could be processed by another DNA glycosylase. As such, we next examined the *ung-1* mutant for responses towards 5-hmU. Unexpectedly, we found that the *ung-1(tm2862)* deletion mutants exhibited decrease viability (nearly 45%), as well as elevated levels of germ cell death upon exposure to 5-hmU (Fig. 5B,F), suggesting that UNG-1 might recognize and remove 5-hmU lesions from the genome. RNAi downregulation of *apn-1* in the *ung-1(tm2862)* mutant further decreased the viability of the animals and enhanced the number of apoptotic germ cells (nearly 80%) upon 5-hmU exposure (Fig. 5B,F), suggesting that both UNG-1 and APN-1 contribute independently to process the 5-hmU lesion. Interestingly, the diminished viability and enhanced germ cell death observed by RNAi downregulation of *apn-1* in the *ung-1(tm2862)* mutant was not seen when similar experiment was conducted in the *nth-1(ok724)* mutant (Fig. 5A,E). We interpret this finding to suggest that NTH-1 might act *in vivo* to remove some of the 5-hmU lesions and at the same time produce toxic 3′-α, β unsaturated aldehyde that requires the 3′-diesterase function of APN-1. As such, in the absence of NTH-1 these toxic 3′-α, β unsaturated aldehyde lesions are not generated.

Since *exo-3(tm4374)* mutants were not sensitive to 5-hmU, we tested whether RNAi downregulation of *ung-1* would alter its sensitivity. Interestingly, the knockdown of *ung-1* in the *exo-3(tm4374)* mutant did not sensitize the *exo-3(tm4374)* mutant to 5-hmU (Figure S5A). This unexpected finding prompted us to check whether *apn-1* gene expression level would be induced in the *exo-3(tm4374)* mutant, and as such, compensates to repair 5-hmU lesions in the absence of UNG-1. Indeed, the *apn-1* expression level was at least 2-fold higher in the *exo-3(tm4374)* mutant as compared to the wild type, and which was stimulated to 3-fold upon treatment of the animals with 5-hmU (Figure S5B). We believe that the lack of sensitivity of the *exo-3(tm4374)* mutant to 5-hmU might be explained by the induction of *apn-1* gene expression.

### Recombinant UNG-1 exhibits 5-hmU activity

**Figure 6:**
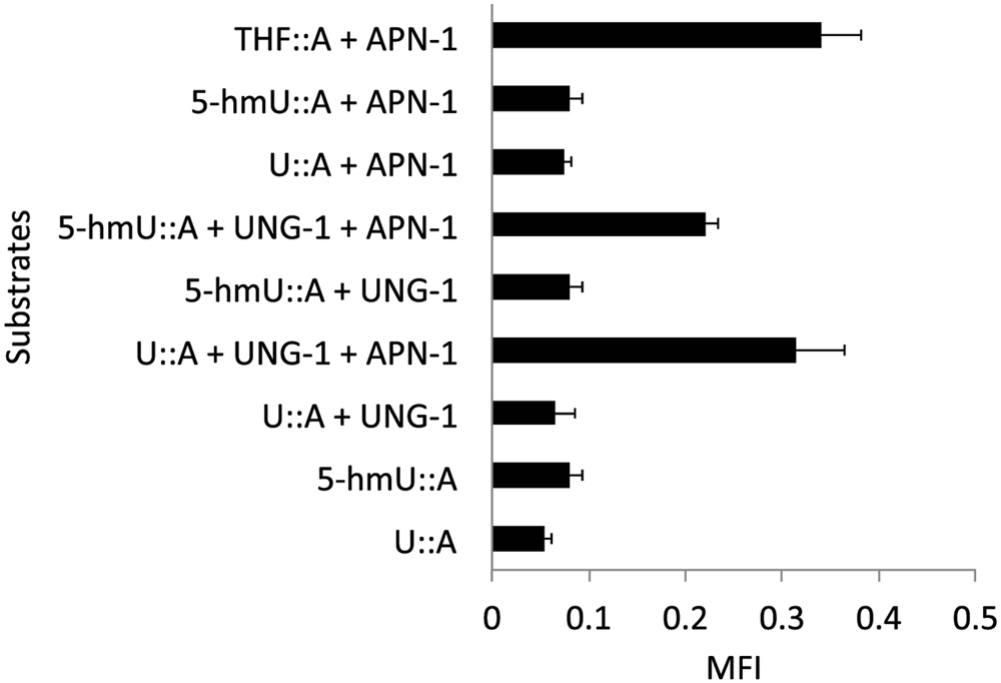
Recombinant GST-UNG-1 acts on 5-hmU lesion to produce APN-1 cleavable AP site. GST-UNG-1 and GST-APN-1 were purified from *E. coli* and *S. cerevisiae* expression system, respectively^14,20^. The enzymes were incubated with the indicated stem-loop deoxyoligonucleotide substrates bearing either uracil, 5-hmU or the AP site tetrahydrofuran (THF) opposite adenine (U:A, 5-hmU:A and THF:A) at the six position from the 5′-end bearing 6-Carboxyfluorescein^35^. UNG-1 was incubated with the substrates for 30 mins at 26°C followed by the addition of APN-1 and then a further incubation for 30 mins at 37°C. The enzymatic incision of the substrate released a fluorescently labeled 5-mer product that can be detected by a fluorometer (Fluoroskan Ascent).

We next checked whether UNG-1 has the ability to remove 5-hmU from an oligonucleotide substrate. To do this, we created stem-loop deoxyoligonucleotide substrates bearing either uracil, 5-hmU or the AP site tetrahydrofuran (THF) opposite adenine (U:A, 5-hmU:A and THF:A) at the six position from the 5′-end bearing 6-Carboxyfluorescein^35^. We incubated the substrates for 30 mins at 25 °C without and with GST-UNG-1 purified from an *E. coli* expression system (Figure S6)^14^ followed by the addition of *C. elegans* APN-1 purified from a *S. cerevisiae* expression system^20^ and then a further incubation for 30 mins at 37 °C. The enzymatic incision of the substrate released a fluorescently labeled 5-mer product that can be detected by a fluorometer. The purified GST-UNG-1 removed uracil from the U:A substrate to create an AP site that was cleaved by the purified GST-APN-1 (Fig. 6). Interestingly, the purified GST-UNG-1 also processed the 5-hmU substrate, which was subsequently cleaved by GST-APN-1 (Fig. 6). In control experiments, GST-UNG-1 alone did not produce the cleaved product from either the U:A or the 5-hmU:A substrate, unless GST-APN-1 was added (Fig. 6), suggesting that the GST-UNG-1 preparation has no contaminating AP endonuclease or AP lyase activity. In additional controls, GST-APN-1 alone did not incise the U:A or 5-hmU:A substrate, unless the substrates were pre-incubated with GST-UNG-1 (Fig. 6). However, GST-APN-1 alone incised the THF substrate (Fig. 6) and not the GST-UNG-1 as determined in other control experiments. These data strongly suggest that UNG-1 possesses the ability to remove 5-hmU, albeit less effectively as compared to uracil (Fig. 6).

### POLQ-1 is required for DNA synthesis after removal of 5-hmU lesions

Upon removal of the 5-hmU lesion and incision of the AP site by the BER pathway, a DNA polymerase would be required to fill the single nucleotide gap with a correct nucleotide^36^. A previous study identified POLQ-1 as the DNA polymerase required for the insertion of single nucleotide in the BER pathway in *C. elegans*^37^. In addition, another study showed that POLQ-1 is involved in the repair of DNA interstrand cross-link (ICL)^38^. Furthermore, *C. elegans* disrupted for the *polq-1* gene showed hyper-activation of the DNA damage checkpoint-dependent cell-cycle arrest, as well as enhanced apoptosis in germ cells following treatment with ICL agents^38^. As such, we reasoned that POLQ-1 could be involved in processing 5-hmU and that its downregulation would leave a single nucleotide gap in the damaged strand, which in turn will trigger germ cell apoptosis. Indeed, we found that the *polq-1(tm2572)* mutants exposed to 5-hmU showed a significant increase in the average number of apoptotic cells (9.0 ± 1.0) as compared to the no treatment condition (5.0 ± 1.0) (Figure S7). Depletion of *apn-1* in the *polq-1(tm2572)* mutant resulting in the *polq-1(tm2572); apn-1(RNAi)* knockdown mutant that showed an increase in the average number of 12.0 ± 2.0 of apoptotic germ cells when treated with 5-hmU, as compared to 8.0 ± 1.0 in the untreated (Figure S7). The data suggest that POLQ-1 performs additional roles besides serving as the DNA polymerase required to fill the single nucleotide gap created following removal of 5-hmU from the genome. In fact, *C. elegans* POLQ-1 is related to the human DNA polymerase *theta* that is responsible for repairing double strand breaks in the alternative NHEJ pathway^39^.

### Mutants defective in either the MMR, NER or HR pathway do not show significant increase in germ cell death upon 5-hmU exposure

We next examined whether processing of the 5-hmU lesions would be specific to the BER pathway or the lesion could be channeled to other DNA repair pathways. To do this, we selected representative mutants of the three additional DNA repair pathways, namely *msh-2* of the mismatch repair (MMR), *xpa-1* of the nucleotide excision repair (NER) and *rad-51* of the homologous recombination (HR) repair pathways and checked for the extent of germ cells apoptosis following exposure to 5-hmU.

**Figure 7:**
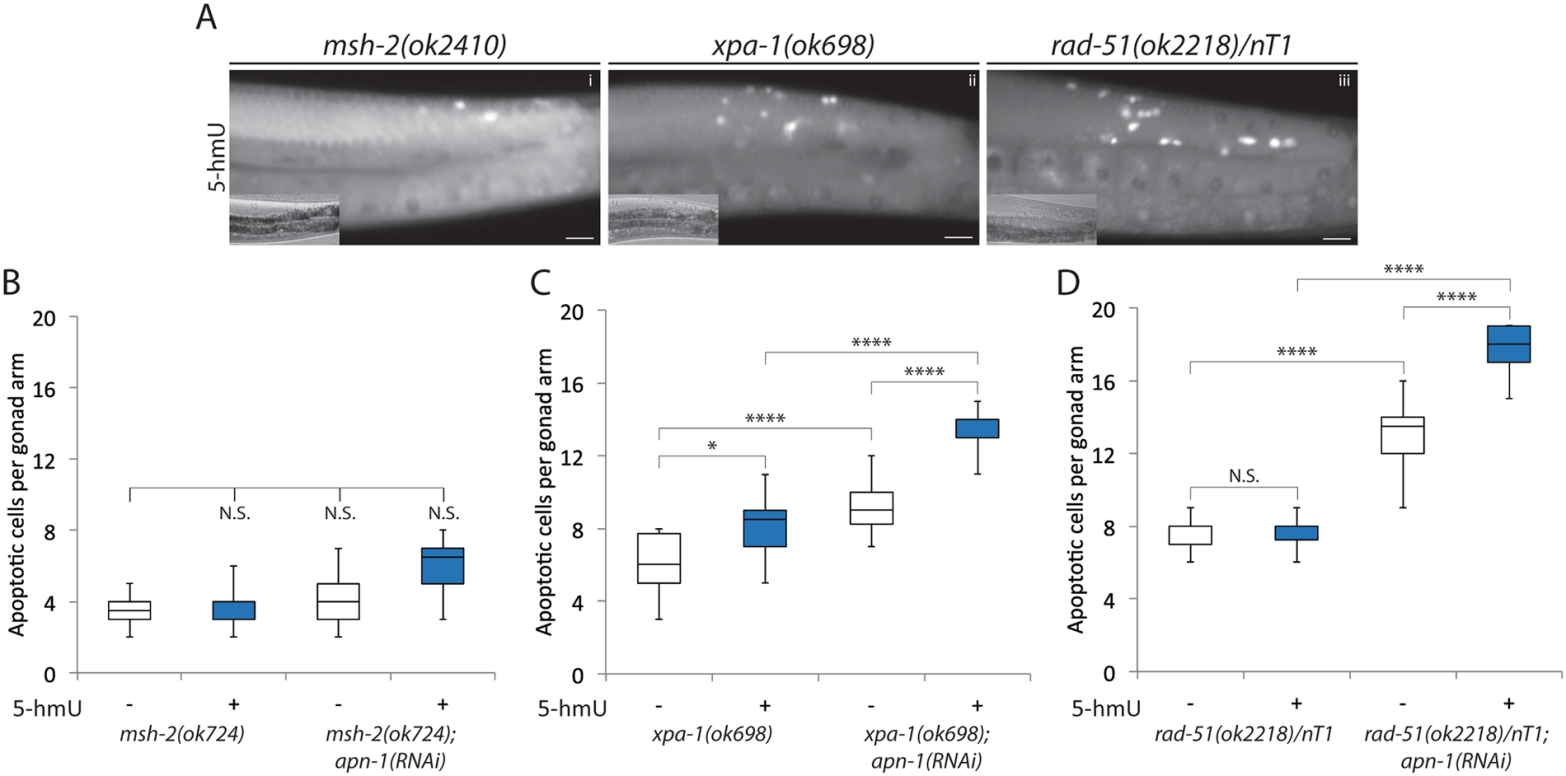
Mutants defective in the MMR, NER or HR pathway do not show significant increase in germ cell death upon exposure to 5-hmU. (**A**) Acridine orange stained and DIC images as described in Fig. 2. The images represent a component from each of the three additional main DNA repair pathways, [i] *msh-2(ok2410)* Mismatch Repair pathway, [ii] *xpa-1(ok698)* Nucleotide Excision Repair pathway and [iii] *rad-51(ok2218)* Homologous Recombination pathway, respectively. (**B**–**D**), Box and whisker plots depicting quantification of apoptotic corpses observed by the DNA repair defective mutants in the absence and presence of 5-hmU treatment.

We first assessed the levels of germ cell apoptosis in the MMR deficient mutant *msh-2(ok2410)*. These *msh-2(ok2410)* mutants did not display significant numbers of apoptotic cells either under standard growth conditions or when treated with 5-hmU (4.0 ± 1.0 and 4.0 ± 1.0, respectively) (Figs 7A[i] and 6B). Downregulation of the *apn-1* gene *via* RNAi in the *msh-2(ok2410)* mutant did not increase the average number of apoptotic cells in the *msh-2(ok2410); apn-1(RNAi)* mutant following 5-hmU exposure (4.0 ± 1.0 and 6.0 ± 2.0, respectively) (Fig. 7B), as compared to level (14.3 ± 1.7) in the treated *apn-1(tm6691)* mutant (Fig. 3B[vi vs. ii] and D). Since it was previously reported that defects in the MMR pathway reduce DNA damage-induced germ cell apoptosis following exposure to genotoxic stress^40^, we interpret our finding to suggest that MSH-2 could act to recognize the 5-hmU lesion and recruits NTH-1. Thus, in the absence of MSH-2, NTH-1 would be blocked from generating the toxic 3′-α, β unsaturated aldehyde lesions in DNA and therefore protects the *apn-1* mutant from 5-hmU.We next examined whether the NER pathway mutant *xpa-1(ok698)* would undergo germ cell apoptosis when challenged with 5-hmU. These *xpa-1(ok698)* mutant animals devoid of the XPA-1 protein showed an increase in the average number of germ cell apoptosis under standard growth conditions and which was slightly elevated after exposure to 5-hmU (6.0 ± 2.0 and 8.0 ± 1.0, respectively) (Fig. 7A[ii] and C). Upon downregulation of apn-1 by RNAi, the resulting *xpa-1(ok698); apn-1 (RNAi)* knockdown mutant displayed higher levels of apoptotic cells upon exposure to 5-hmU, and depicting the same average number (14.0 ± 1.0) of apoptotic cells per animal (Fig. 7C), as the treated *apn-1(tm6691)* mutant (Figs 3B[vi vs. ii] and 2D), excluding a major role for the NER pathway in processing the 5-hmU lesion.

We finally examined the involvement of the HR pathway in processing the 5-hmU lesion using the *rad-51(ok2218)/nT1* mutant. This rad-51 mutant already showed high endogenous levels of germ cell apoptosis due to spontaneous unrepaired meiotic breaks as evidenced by an average number of apoptotic cells of 8.0 ± 1.0 under standard growth conditions (Fig. 7D). However, the number of germ cell corpse did not increase upon exposure to 5-hmU (8.0 ± 1.0) (Fig. 7A[iii] and D), suggesting that the 5-hmU lesion is this mutant is processed by other dominant DNA repair pathway. We therefore depleted *apn-1* expression via RNAi in the *rad-51*(*ok2218*)/*nT1* mutant and the resulting *rad-51(ok2218)/nT1*; *apn-1(RNAi)* knockdown mutant showed a substantial increase in the average number of apoptotic cells after treatment with 5-hmU (18.0 ± 1.0) (Fig. 7D). Thus, it would appear that the high level of germ cell death in the *rad-51(ok2218)/nT1*; *apn-1(RNAi)* mutant can be explained by an additive effect from the spontaneous germ cell death from the *rad-51* mutant and that caused by the lack of APN-1 to process the 5-hmU lesions. Taken together, we conclude that BER is the predominant pathway involved in processing the 5-hmU lesions, while the NER pathway may have a minor role and not that of the MMR and HR pathways.Discussion

In this study, we present several novel findings regarding the base-excision repair pathway in *C. elegans* that is equipped with two DNA glycosylases, UNG-1 and NTH-1, as well as two AP endonucleases/3′-diesterases, APN-1 and EXO-3, to remove damaged bases and process the resulting AP sites^4,13,15^. *C. elegans* UNG-1 has been shown to remove uracil, while NTH-1 has a broader substrate specificity and removes thymine glycol, 5-formyluracil and 5-hmU from lesion containing DNA substrates using purified enzymes^13–15^. In the case of the two AP endonuclease/3′-diesterases, we have previously shown that APN-1 has additional enzymatic activities and can act on many types of DNA lesions *in vitro*, while this ability is restricted for EXO-3^4^. Based on the *in vitro* specificities of these enzymes one might expect that *C. elegans* devoid of both NTH-1 and APN-1 would have the most severe phenotypes when challenged with DNA damaging agents. Herein, we challenged *C. elegans* BER-deficient mutants with the nucleoside form of 5-hmU, and unexpectedly observed very striking phenotypes that prompted a reconsideration of the *in vivo* roles of UNG-1 and NTH-1 in this organism. In our approach, we exposed the animals to 5-hmU and monitored several readouts including brood size, lifespan and germ cell apoptosis. The latter analysis is a very sensitive reporter especially for agents that induce genotoxic stress and mutants defective in DNA repair exhibit elevated levels of germ cell death. The observations that 5-hmU caused a decrease in the brood size and lifespan, as well as an increase in germ cell apoptosis, prompted the conclusion that 5-hmU must be incorporated into the genome of the animals to trigger a DNA damage response. In fact, mass spectrometry analysis revealed that if mammalian cells were exposed to 5-hmU this oxidized nucleoside became incorporated into the genome^32^. We attempted to monitor the levels of 5-hmU lesion in *C. elegans* genome, but we were unable to observe a significant and consistent increase in BER deficient mutants. Further studies are in progress to examine the incoporation and repair of 5-hmU in the genomic DNA of *C. elegans.* Nonetheless, we have partially purified recombinant UNG-1 as a GST fusion protein and demonstrated that it has the ability to act on 5-hmU lesion installed on a stem-loop deoxynucleotide substrate. This finding is consistent with the *ung-1* mutants being very sensitive to 5-hmU exposure. In fact, we were surprised that the *nth-1* mutants showed very little or no sensitivity towards 5-hmU, although NTH-1 was previously shown to possess the ability to remove 5-hmU from lesion containing DNA substrates *in vitro* ^13–15^. We propose that *C. elegans* UNG-1 may have evolved to acquire a broader substrate specificity and thus could act as the dominant DNA glycosylase *in vivo* to remove various modified forms of uracil such as the 5-hmU lesion. It is noteworthy that *C. elegans* lacks the related human SMUG1 DNA glycosylase, which has been shown to remove 5-hmU^6^, and the *C. elegans* UNG-1 shares a modest 12.6% identity with SMUG1^32^. A closer examination of the identity revealed that *C. elegans* UNG-1 shares five amino acid residues Ser58, Pro218, Gly226, Glu233 and Leu234 that are unique to human SMUG1 residues Ser48, Pro166, Gly174, Glu181 and Leu182 and the mouse SMUG1, but which are absent in human UNG1 (Figure S8). Whether these five residues are involved in conferring upon *C. elegans* UNG-1 the human SMUG1 ability to recognize and process 5-hmU will need to be investigated.

**Figure 8:**
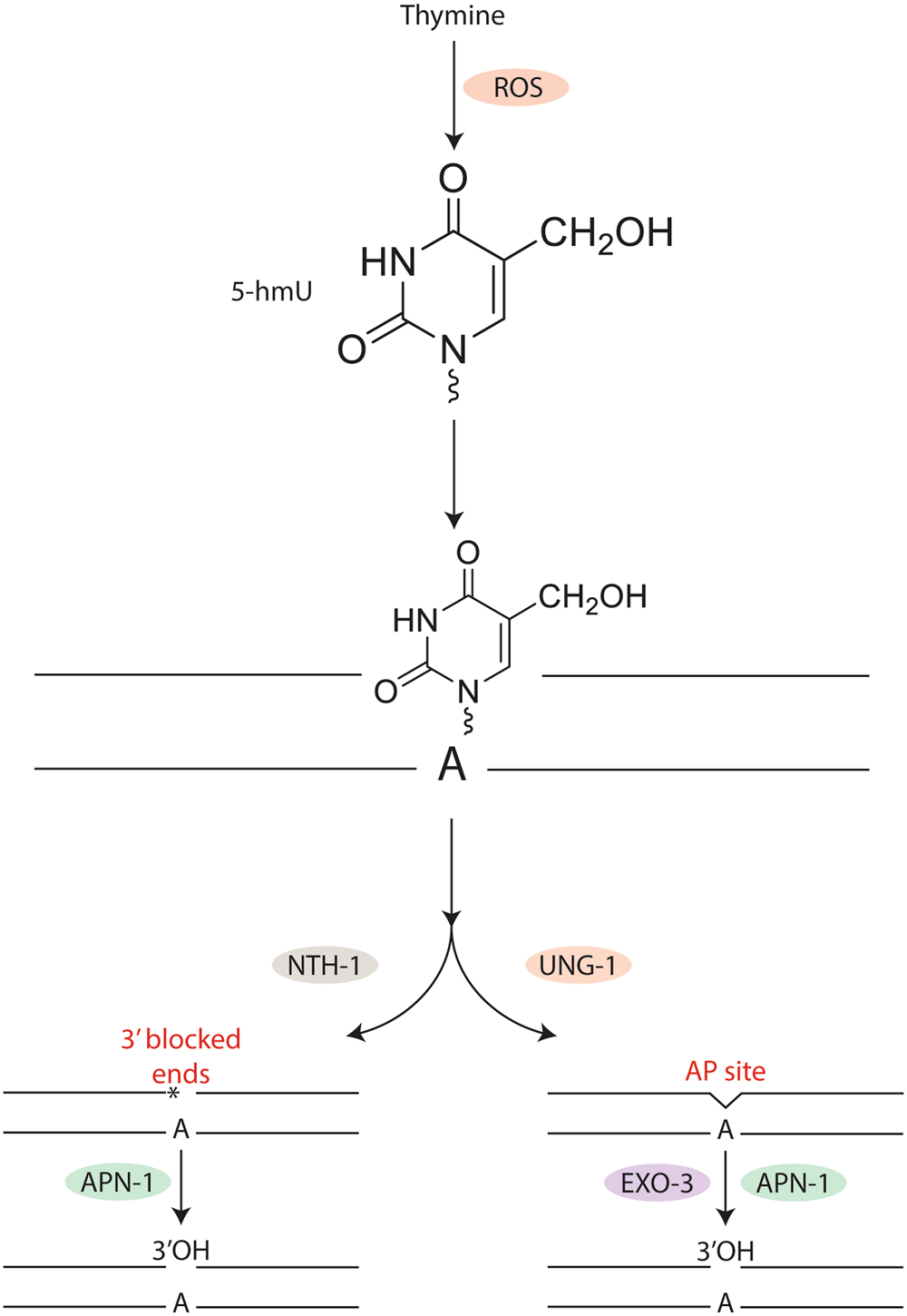
A model illustrating the repair of 5-hmU opposite adenine (5-hmU•A) *via* the BER pathway. Under physiological conditions thymine in the genome is oxidized to 5-hmU creating the mispair 5-hmU•A. In this model, UNG-1 is proposed to recognize and remove 5-hmU creating an AP site that can be processed by either APN-1 or EXO-3. In the absence of UNG-1, NTH-1 removes the 5-hmU lesion and simultaneously cleaves the resulting AP site to create a secondary 3′-blocked genotoxic lesion, 3′-α, β unsaturated aldehyde, which requires processing by the 3′-diesterase or 3′- to 5′-exonuclease activity of APN-1. The 5-hydroxymethyluracil (5-hmU) structure was obtained from PubChem (ID: 78168) and rendered with Chemaxom.

UNG-1 action on 5-hmU would leave an AP site that can be processed by either APN-1 or EXO-3 (see model in Fig. 8). If the AP endonuclease function of APN-1 and EXO-3 is redundant in *C. elegans*, then the absence of either enzyme would not cause sensitivity to 5-hmU. Since our data revealed that (i) *C. elegans* mutants devoid of APN-1 were sensitive to 5-hmU, but not EXO-3 deficient animals, (ii) *apn-1*, and not *exo-3*, gene expression was inducible upon treatment of the animals with 5-hmU, and (iii) *apn-1* gene expression is constitutively higher in the *exo-3* mutant, we strongly suggest that APN-1 has a vital role in processing the 5-hmU lesions. In fact, we have previously reported that APN-1 has distinct function(s) from EXO-3, and more recently SenGupta *et al*., 2013 showed that accumulation of the early DNA damage response foci, RPA-1, following treatment with the anticancer agent 5-fluorouracil, depends primarily on EXO-3 role to incise the DNA in order to initiate the mismatch repair pathway^18,41^. We propose a model whereby UNG-1 removes 5-hmU creating an AP site that is either processed by APN-1 or EXO-3^23,42^ (Fig. 8). In the absence of UNG-1, the 5-hmU lesion is processed by the second pathway whereby NTH-1 removes the lesion, but simultaneously creates a genotoxic single strand break with a blocked 3′-end, α,β unsaturated aldehyde that blocks DNA repair synthesis^7^. It is possible that the resulting blocked 3′-end is not rapidly removed by APN-1, in particular, if EXO-3 stimulates NTH-1, as seen for the DNA glycosylases OGG1 and TDG by APE1 in human cells^43,44^, to create an abundance of blocked 3′-ends that overwhelms the 3′-diesterase and or 3′- to 5′-exonuclease repair capacity of APN-1. Therefore, animals devoid of both UNG-1 and APN-1 would be extremely sensitive to 5-hmU due to excessive accumulation of single strand breaks with blocked 3′-ends. If indeed the *apn-1* mutant sensitivity to 5-hmU is a result of NTH-1 β-lyase activity generating the production of single strand breaks with blocked 3′-ends, then the removal of NTH-1 should rescue the lethality of the *apn-1* mutant. In fact, we showed herein that RNAi downregulation of *nth-1* can suppress the genotoxic effects of 5-hmU in the *apn-1* mutants. A similar suppressive effect was also observed in the *msh-2*; *apn-1(RNAi)* knockdown mutant, which may implicate MSH-2 in the same pathway as NTH-1. Thus, in the absence of NTH-1 and APN-1, as in the *nth-1(ok724)* mutant downregulated for *apn-1* by RNAi, the 5-hmU lesion would be removed by UNG-1 leaving an AP site that will be processed by the AP endonuclease activity of EXO-3^23,42^. We exclude the possibility that EXO-3 acts to remove the 3′- α, β unsaturated aldehyde generated by NTH-1, as the *exo-3(tm4374); apn-1(RNAi)* knockdown mutant is no more sensitive to 5-hmU than the *apn-1(tm6691)* single mutant. Consistent with this notion, we have previously shown that EXO-3, unlike APN-1, lacks a 3′- to 5′-exonuclease activity, which might be the activity needed to remove such bulky and toxic 3′-blocked end^18–21^. In fact, it has been reported that diminishing UNG-1 activity to prevent production of AP sites recues the lifespan defect of the *exo-3* mutants^23^. This phenomenon was not observed in *exo-3* mutants devoid of NTH-1, suggesting that this DNA glycosylase is not producing intermediate lesions to be processed by EXO-3^23^.

In short, we have established that the oxidized product of thymine, 5-hmU, is genotoxic in *C. elegans* and that this lesion is predominantly processed by the BER pathway. The most striking observation from our study is the requirement of UNG-1 for the removal of 5-hmU. We believe that *C. elegans* may have conserved only UNG-1 and NTH-1, which have evolved to recognize a broad spectrum of modified bases.

## Materials and Methods

### Nematode strains and culture conditions

The [*apn-1(tm6691)* II], [*exo-3(tm4374)* I], [*ung-1(tm2862)* III] and [*polq-1(tm2572)* III] were obtained from Shohei Mitani (Tokyo Women′s Medical University School of Medicine, Japan and the National Bioresource Project for the nematode *C. elegans*). The Bristol N2 (wild type), MD701 [*bcIs39* [lim-7p::ced-1::GFP + lin-15( + )], RB877 [*nth-1(ok724)* III], RB1864 [*msh-2(ok2410)* I], RB864 [*xpa-1(ok698)* I], VC1973 [*rad-51(ok2218) IV/nT1* [qIs51] (IV;V)] *, TJ1 [*cep-1(gk138)* I], MT1082 [*egl-1(n487)* V], MT4770 [*ced-9(n1950)* III], MT5287 [*ced-4(n1894)* III], MT3002 [*ced-3(n1286)* IV] *Caenorhabditis elegans* strains were obtained from the CGC Stock center (*Caenorhabditis* Genetics Centre, University of Minnesota, Minneapolis, USA). The alleles utilized in this work were all previously validated to be null. All *C. elegans* strains were maintained at 20 °C on nematode growth medium (NGM) agar (2.5 g/L peptone, 51.3 mM NaCl, 17 g/L agar, 1 mM CaCl_2_, 1 mM MgSO_4_, 25 mM KPO_4_, and 12.9 μM cholesterol) enriched with a lawn of streptomycin-resistant *Escherichia coli* OP50 bacterial strain as a source of food. For all *in vivo* experiments, developmental staged-synchronized nematodes were obtained by hypochlorite treatment of gravid adult hermaphrodites. Eggs were allowed to hatch on M9 buffer (6 g Na_2_HPO_4_, 3 g KH_2_PO_4_, 5 g NaCl, 0.25 g MgSO_4_•7H_2_O per liter filter sterilized). In all experiments, animals were monitored from day 1 post-L1 larvae stage and from L4 to avoid experimental bias. *Homozygous *rad-51/rad-51*^45^ animals show almost complete inviability due to high embryonic lethality in their progeny, therefore we monitored heterozygote animals due to the easy RNAi-feeding for further analyses. *C. elegans* strains were backcrossed at least three times.

### Drug treatment

For drug treatment we followed the same protocol as recently reported^28^. The anthracycline doxorubicin, alkylating agent methyl methanesulfonate (Sigma Cat. No 129925), the oxidative agent 5-hydroxymethyluracil and the water-soluble platinum compound cisplatin were added to the NGM agar medium (55 °C) before solidification to obtain a final concentration of 100 μM for doxorubicin and cisplatin, 0.25 μM for methyl methanesulfonate and 1 μM for 5-hydroxymethyluracil (molecular weight 258 g/mole), respectively. For all experiments, L1-staged from F1 synchronized nematodes were transferred to NGM control agar plates and containing doxorubicin, cisplatin and methyl methanesulfonate. Doxorubicin and cisplatin working concentrations were chosen based on previously reported assays^46^. All drug-containing plates were freshly made prior to each experiment. 5-hydroxy-2′-deoxyuridine (5-hmU) was prepared as previously described^47^. The oncology pharmacy department of the Maisonneuve-Rosemont Hospital (HMR), provided doxorubicin and cisplatin.

### Brood size analyses

Single L1-staged worm from wild type and mutant genotypes were transferred to seeded NGM plates without and with the drugs and maintained at 20 °C. Worms were transferred to fresh plates each day until they ceased laying eggs. The hatched larvae on each plate were counted and total number of viable larvae that developed to the L1 stage descended from a single hermaphrodite was counted. The average number of viable larvae from 10 to 25 animals of a strain was plotted as brood size where the progeny is allowed to reach adulthood and scored as being fertile or sterile. The brood size quantification in this analysis follows the same method as previously reported^28^.

### Lifespan assay

Lifespan analyses were performed at 20°C in standard conditions and assessed blindly as previously reported^48^.

### Microscopy and imaging

All microscopy was performed utilizing a DeltaVision Elite Image Restoration System (Applied Precision) with either 40 × /0.65–1.35 or 63 × /1.42 oil objective. The worms were anesthetized with levamisole (5 μM, Sigma Cat. No L0380000) and mounted on 2% agarose pads for their respective imaging and quantification. Images were processed utilizing ImageJ imaging software^49^.

### DNA damage response assay and germ cells imaging

The methods previously described were used^28,30^. Briefly, for scoring of apoptotic corpses in nematodes, L1-staged synchronized N2 wild type and DNA repair deficient mutants were exposed to different doses of drugs followed by germ cells apoptosis assay. Between 18 to 24 hours past L4-staged nematodes, adult staged worms were assayed with differential interference contrast (DIC) microscopy (Nomarski) optics and the vital DNA dye acridine orange (Sigma Cat. No A6024). Nematodes were incubated in the dark for 2 hours at 20 °C on NGM plates containing 1 ml of 50 μg/ml of acridine orange DNA dye dissolved in M9 buffer. Stained worms were transferred to fresh OP50-seeded NGM plates to incubate for 2 hours in order to clear off the stained bacteria. The acridine orange-stained and DIC-visible apoptotic corpses were counted with an exposure time of 1 second and 0.8 seconds, respectively. The engulfment of apoptotic corpses was scored utilizing the CED-1::GFP reporter and imaged similarly with an exposure time of 1 second utilizing the GFP channel. Images were collected as a series of 25/0.5 μm optical sections covering the complete thickness of the gonad arm.

### Purification of recombinant GST-UNG-1

The plasmid pGEX-CeUNG-1 designed to express UNG-1 as a GST-UNG-1 fusion protein was kindly provided by Dr. Qiu-Mei Zhang-Akiyama (Japan). The plasmid was introduced into the *E. coli* strain BL21(DE3) and ampicillin resistant colonies were used for preparing whole cell extracts derived from 50 ml of cells (OD 600 of 0.6 treated with 0.1 mM IPTG for 12 hours to induce the expression level of GST-UNG-1) for the purification of the protein as previously described, except using GST-magnetic beads^14^. The GST-magnetic beads (50 µl) were washed three times with washing buffer (PBS plus 125 mM Tris-HCl pH 7.5 and 150 mM NaCl), incubated with 500 µl of whole cell extract for 2 hours at 4oC, following three washes with 200 µl of washing buffer and elution with three 100 µl of elution buffer (washing buffer containing 50 mM glutathione).

### Preparation of oligonucleotide substrates and assay conditions

The preparation of the substrates and assay conditions were as previously described^35^, except GST-UNG-1 was pre- incubated with the substrate in the BER reaction buffer 25 mM HEPES pH 8.0, 150 mM KCl, 0.5 mM EDTA pH 8.0, 1% glycerol and 1 mM DTT (prepared fresh) for 30 mins at 25°C, prior to the addition of the reaction buffer (50 mM HEPES pH 7.5, 50 mM KCl and 10 mM MgCl_2_) and a further incubation with purified GST-APN-1 for 30 mins at 37°C. The reactions were carried out, where indicated, with 20 ng of purified GST-UNG-1 and 20 ng of purified GST-APN-1 and the released product was monitored by a fluorometer (Thermo Fisher Scientific, Thermo Scientific^TM^, model: Fluoroskan Ascent^TM^).

### Relative RNA quantification to monitor gene expression

Total RNA (RNeasy mini kit Qiagen Cat. N° 74104) was prepared from ~1000 young adult synchronized nematodes and used for cDNA synthesis (Applied Biological Materials Inc. Cat. N° G490) followed by quantitative real-time PCR (qRT-PCR). qRT-PCR was performed with the BrightGreen 2 × q-PCR Mastermix (Applied Biological Materials Inc. Cat. N° MasterMix-LR) starting at 95 °C for 2 min, followed by 40 cycles at 95 °C for 5 sec, 60 °C for 30 sec and 72 °C for 30 sec. Transcript levels were normalized to the internal control *act-1* encoding the actin protein. The forward and reverse primer sequences utilized in this study were: *apn-1*: 5′-GCACATCCAGAAGACGCTGC-3′ and 5′-TCTACGGTAGTTCCAGGGCT-3′; *exo-3*: 5′-AGGAGCCTGACCTCGTTTTT-3′ and 5′-GTAGCCACCGTTCTTCTCTG-3′; *nth-1*: 5′-TTTCCAGTCAAACCAGAGAT-3′ and 5′-AAATCCAACAGGACACAAAA-3′; *ung-1*: 5′-TTCCGGACATGTTCCTCAAA-3′ and 5′-TTCATTGCCCGCGGGAACTT-3; *act-1*: 5′-TGCTGATCGTATGCAGAAGG-3′ and 5′-TAGATCCTCCGATCCAGACG-3′.

### RNA interference analysis

*Escherichia coli* HT115DE3 strain harboring specific RNAi constructs against *apn-1* (T05H10.2 AAB39924 10018 G6), *exo-3* (R09B3.1 AAC82328 10018 F7), *nth-1* (R10E4.5 R10E4.5 11002 D5) and *ung-1* (Y56A3A.29 Y56A3A.29 10056 D12) was grown on lysogeny broth (LB) agar plates containing ampicillin and tetracycline. Overnight cultures were grown in LB media containing ampicillin. For *apn-1*, *exo-3*, *nth-1* and *ung-1* RNAi-driven knockdown experiments, nematodes were maintained until first generation (F1) on NGM agar plates containing 1 mM IPTG (isopropyl-β-D-1-thiogalactopyranoside) enriched with a lawn of *E. coli* HT115DE3 expressing RNAi constructs in the pL4440-feeding vector at standard temperature 20 °C. For *apn-1*, *exo-3*, *nth-1* and *ung-1* RNAi-driven knockdown efficiency, mRNA expression levels were measured in synchronized young adults collected from the F1 generation of nematodes fed with *E. coli* expressing RNAi targeted to the indicated genes. The RNAi clones were obtained from the Ahringer laboratory library^50^ and verified by sequencing. The depletion efficiency of *apn-1*, *exo-3*, *nth-1* and *ung-1* genes was validated by qRT-PCR. In all experiments synchronized L4-staged animals were fed RNAi expressing bacteria and the resulting F1 animals were analyzed for phenotypes.

### Statistical analyses

For the Brood size analysis, statistical differences were calculated by the unpaired two-tail t-test (*P < 0.03; **P < 0.01; ***P < 0.0005) and represented as ± S.D. Lifespan analyses were performed utilizing the Kaplan-Meier estimator calculating the Log-rank test for statistical significance utilizing OASIS software (Online Application for the Survival Analysis of Lifespan Assays Performed in Aging Research)^51^. Germ cells death statistical significance was assessed with the Mann-Whitney U-test calculator Mean values ± s.e.m were calculated for each condition. *P < 0.05; **P < 0.01; ***P < 0.001; ****P < 0.0001 were considered to be statistically significant. N.S. = Non-Significant. Statistical differences were calculated by using the GraphPad Prism Statistical Software Mac Version 6.

## Electronic supplementary material

Supplemental Figures S1-S8
