# Supplementary material for: UNG-1 and APN-1 are the major enzymes to efficiently repair 5-hydroxymethyluracil DNA lesions in C. elegans
Source: Sci Rep. 2018 May 1;8:6860. doi: 10.1038/s41598-018-25124-1 (PMC5931555; doi:10.1038/s41598-018-25124-1)
Supplement: Supplementary file 1 — Supplemental Figures S1-S8 [file 41598_2018_25124_MOESM1_ESM.pdf]

Supplemental information
Title: UNG-1 and APN-1 are the major enzymes to efficiently repair 5-hydroxymethyluracil DNA lesions in *C. elegans*

Arturo Papaluca, J. Richard Wagner, Uri Saragovi and Dindial Ramotar

Legends for Supplemental Figures

Figure S1. Sequence alignment of DNA glycosylases (A) CeNTH-1 and hNTH1 and (B) CeUNG-1 and hUNG1 belonging to *C. elegans* and *H. sapiens* respectively. Numbers indicate amino acid positions. Identical or similar amino acid residues amongst the sequences are shaded in black or gray, respectively. Dashes indicate gaps. Sequence similarity: **(A)** 67.4 % and **(B)** 58.2 %.

Figure S2. A, The relative gene expression of the *apn-1*, *exo-3*, *nth-1* and *ung-1* transcripts in the wild type and the respective deletion mutant animals *apn-1(tm6691)*, *exo-3(tm4374)*, *nth-1(ok724)* and *ung-1(tm2862).* RNAi-driven depletion was measured and corrected on actin as an internal control. Synchronized young adult animals were collected and mRNA levels were assessed by qRT-PCR. Data shown represent the average ± s.d. from a 60 × 15 mm petri dish of animals (n ~ 1000) pooled from three independent experiments. **B**, **5-hmU induces the expression of the *apn-1*, *nth-1* and *ung-1* genes, but not the *exo-3* gene**. The animals were treated for 5 hours with 5-hmU (1 µM) and *apn-1* mRNA assessed by qRT-PCR. **C**, **The expression pattern of the indicated genes during various developmental stages of *C. elegans*.** The data were obtained from the Wormbase.

Figure S3. Kaplan-Meier survival plot of BER components exposed to DNA damaging agents. A, B and C, Kaplan-Meier survival plot showing alive percentage of the indicated genotypes. L1-staged animals (*n*=100) were exposed to **A**, No treatment, **B**, DOX and **C**, MMS. Lifespan was blindly assessed starting from young adult animals. The mean lifespan of two independent experiments is shown.

Figure S4. Genetic analysis of control and 5-hmU-induced apoptotic germ cell death. A, Representative chart showing the average quantification of germ cell death in

three independent experiments (*n*=30) corresponding to the wild type and the apoptotic defective mutants *cep-1*, *egl-1*, *ced-9*, *ced-4* and *ced-3* without and with RNAi-driven downregulation of *apn-1* in the absence and presence of 5-hmU (1 μM). Apoptotic cell corpses were observed and scored as described in the experimental procedures.

Figure S5. A, *exo-3(tm4374)* mutants are not sensitized to 5-hmU upon ung-1 downregulation by RNAi. Brood size analyses of the indicated genotypes. The data are the mean±S.D. of three independent experiments (*n*=10 monitored for 3 days). Control, Wild type; *exo-3(tm4374)*; and *exo-3(tm4374); ung-1(RNAi).* **B, *apn-1* expression level in the wild type and *exo-3(tm4374)* mutant without and with 5-hmU exposure.** The animals were untreated or treated for 5 hours with 5-hmU (1 μM) and *apn-1* mRNA assessed by qRT-PCR.

Figure S6. Recombinant GST-UNG-1 purified from an *E. coli* expression system. BL21(DE3) expressing *C. elegans* UNG-1 as a GST-UNG-1 fusion protein was purified according to Nakamura *et al*., 2008, but using GST-magnetic beads. WCE, whole cell extract, F-Th, flow through from the GST-magnetic beads, E1, E2 and E3 are the elution fractions.

Figure S7. POLQ-1 is required for DNA synthesis after removal of 5-hmU lesions. **A**, Representative images of the posterior gonad arms of the *polq-1* mutant stained with acridine orange upon treatment with 5-hmU as described in Figure 2. **B**, Box and whisker plots showing quantification of germ cell apoptosis in *polq-1(tm2572)* and *polq-1(tm2572); apn-1*(*RNAi*) in the absence and presence of 5-hmU.

Figure S8. Sequence alignment of the DNA glycosylases CeUNG-1, hUNG1 and hSMUG1 belonging to *C. elegans* and *H. sapiens*, respectively. Numbers indicate amino acid positions. Identical or similar amino acid residues amongst the sequences are shaded in black or gray, respectively. Dashes indicate gaps. The residues in green are unique to CeUNG-1 and hSMUG1, but not hUNG1.

Title: UNG-1 and APN-1 are the major enzymes to efficiently repair 5-hydroxymethyluracil DNA lesions in *C. elegans*

Arturo Papaluca, J. Richard Wagner, Uri Saragovi and Dindial Ramotar

A

```
CeNTH-1      1 MKRVVASSSVAA----------------VATCDVEG------------------------
hNTH1        1 MCSPQESGMTALSARMLTRSRSLGPGAGPRGCREEPGPLRRREAAAEARKSHSPVKRPRK
consensus    1 M     S   A sarmltrsrslgpgag   C  E gplrrreaaaearkshspvkrprk

CeNTH-1     21 ---------------------------TVVAWRRDVELIRKMRKDMIAPVDTMGCHKLAD
hNTH1       61 AQRLRVAYEGSDSEKGEGAEPLKVPVWEPQDWQQQLVNIRAMRNKKDAPVDHLGTEHCYD
consensus   61 aqrlrvayegsdsekgegaeplkvpvw    W   v  IR MR    APVD mG  k  D

CeNTH-1     54 PLAAPPVHRFQVLVALMLSSQTRDEVNAAAMKRLKDHGLSIGKILEFKVPDLETILCPVG
hNTH1      121 SSAPPKVRRYQVLLSLMLSSQTKDQVTAGAMQRLRARGLTVDSILQTDDATLGKLIYPVG
consensus  121   A P VhRfQVLv LMLSSQTrD V AaAM RLk hGLsi  IL      L  il PVG

CeNTH-1    114 FYKRKAVYLQKTAKILKDDFSGDIPDSLDGLCALPGVGPKMANLVMQIAWGECVGIAVDT
hNTH1      181 FWRSKVKYIKQTSAILQQHYGGDIPASVAELVALPGVGPKMAHLAMAVAWGTVSGIAVDT
consensus  181 Fyk K  Yl  T  IL    f GDIP Sl  L ALPGVGPKMA L M iAWG   GIAVDT

CeNTH-1    174 HVHRISNRLGWIK--TSTPEKTQKALEILLPKSEWQPINHLLVGFGQMQCQPVRPKCGTC
hNTH1      241 HVHRIANRLRWTKKATKSPEETRAALEEWLPRELWHEINGLLVGFGQQTCLPVHPRCHAC
consensus  241 HVHRI NRL W KkaT tPE T  ALE  LPk  W  IN LLVGFGQ  C PVrPkC  C

CeNTH-1    232 LCRFTCPSSTAKNVKSETEETSTSIEVKQEVEDEFEDEKPAKKIKKTRKTRTKIEVKTES
hNTH1      301 LNQALCPAAQGL------------------------------------------------
consensus  301 L    CP   a nvkseteetstsievkqevedefedekpakkikktrktrtkievktes

CeNTH-1    292 ET
hNTH1          --
consensus  361 et
```

B

```
ceUNG-1      1 MSKTVRIPDMFLKASAASKRKSAS------------------NTENIP-EKVPAGNENQE
hUNG1        1 MIGQKTLY-SFFSPSPARKRHAPSPEPAVQGTGVAGVPEESGDAAAIPAKKAPAGQEEPG
consensus    1 M     i d F   S A KRk  Spepavqgtgvagvpeesg    IPa K PAGnE

ceUNG-1     42 VKKMKLQAPEPTEIL--------------LKSILTGESWSKLLEEEFKKGYISKIEKFIN
hUNG1       60 TPPSSPLSAEQLDRIQRNKAAALLRLAARNVPVGFGESWKKHLSGEFGKPYFIKLMGFVA
consensus   61          E  e lqrnkaaallrlaar   l   GESW K L  EF K Y  Ki  Fl

ceUNG-1     88 SEVNKGKQVFPPPTQIFTTFNLLPFDEISVVIIGQDPYHDDNQAHGLSFSVQKGVKPPPS
hUNG1      120 E-ERKHYTVYPPPHQVFTWTQMCDIKDVKVVILGQDPYHGPNQAHGLCFSVQRPVPPPPS
consensus  121  e  K   VfPPP QiFT  nl    ei VVIiGQDPYH  NQAHGL FSVQk V PPPS

ceUNG-1    148 LKNIYKELESDIEGFKRPDHGNLLGWTRQGVFMLNATLTVRAHEANSHAKIGWQTFTDTV
hUNG1      179 LENIYKELSTDIEDFVHPGHGDLSGWAKQGVLLLNAVLTVRAHQANSHKERGWEQFTDAV
consensus  181 L NIYKEL sDIE F rP HG L GW rQGV mLNA LTVRAH ANSH   GW  FTD V

ceUNG-1    208 IRIISRQSEKPIVFLLWGGFAHKKEELIDTKKHVVIKTAHPSPLSA-RKWWGCKCFSKCN
hUNG1      239 VSWLNQNS-NGLVFLLWGSYAQKKGSAIDRKRHHVLQTAHPSPLSVYRGFFGCRHFSKTN
consensus  241 i  i  qSe  iVFLLWG fA KK   ID KkH Vi TAHPSPLS yR wwGCk FSK N

ceUNG-1    267 TELENSGRNPINWADL
hUNG1      298 ELLQKSGKKPIDWKEL
consensus  301   L  SGr PI W dL
```

Figure S1. Sequence alignment of DNA glycosylases (A) CeNTH-1 and hNTH1 and (B) CeUNG-1 and hUNG1 belonging to *C. elegans* and *H. sapiens* respectively. Numbers indicate amino acid positions. Identical or similar amino acid residues amongst the sequences are shaded in black or gray, respectively. Dashes indicate gaps. Sequence similarity: **(A)** 67.4 % and **(B)** 58.2 %.

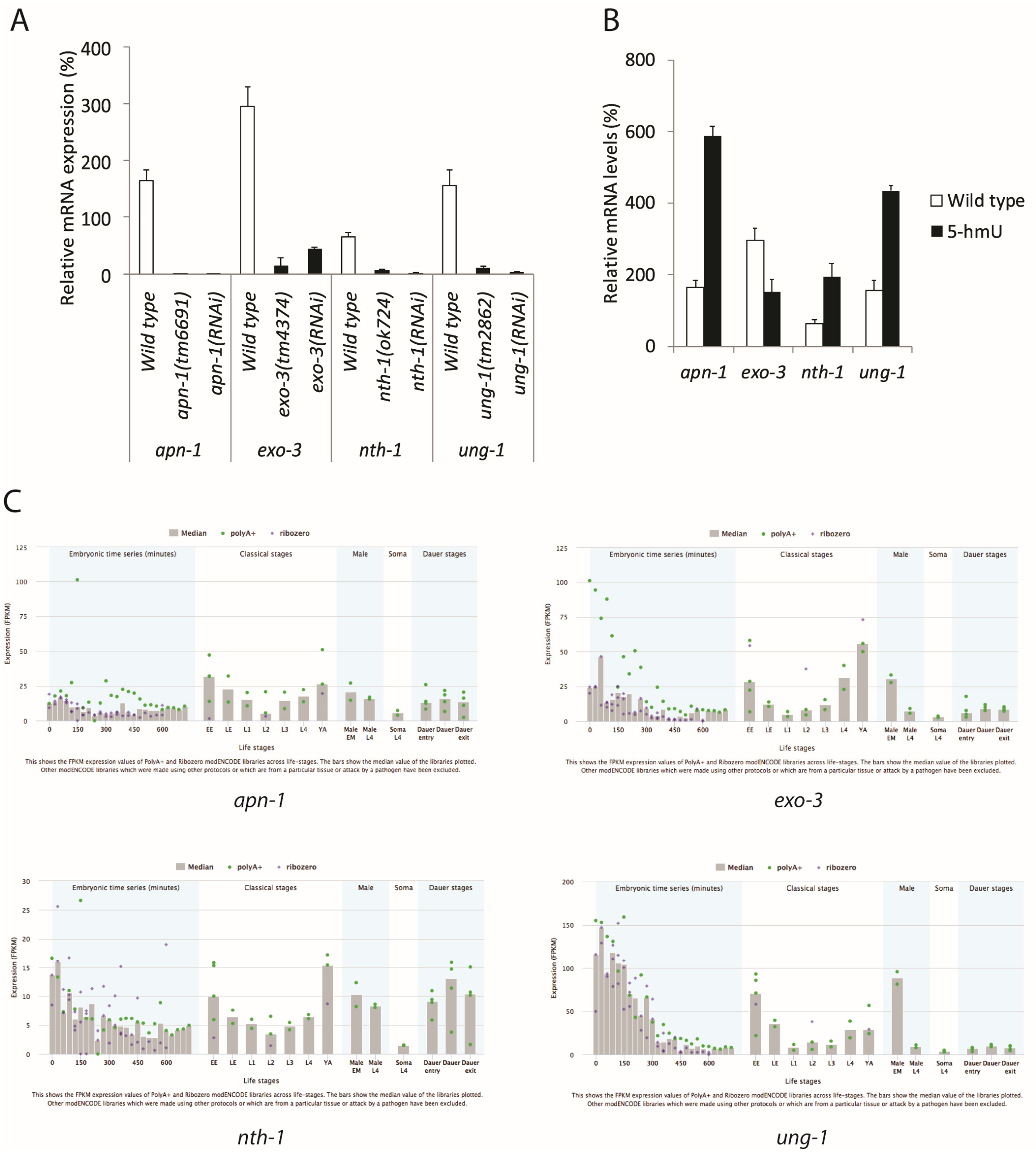

Figure S2. A, The relative gene expression of the *apn-1*, *exo-3*, *nth-1* and *ung-1* transcripts in the wild type and the respective deletion mutant animals *apn-1(tm6691)*, *exo-3(tm4374)*, *nth-1(ok724)* and *ung-1(tm2862)*. RNAi-driven depletion was measured and corrected on actin as an internal control. Synchronized young adult animals were collected and mRNA levels were assessed by qRT-PCR. Data shown represent the average ± s.d. from a 60 × 15 mm petri dish of animals (n ~ 1000) pooled from three independent experiments. **B, 5-hmU induces the expression of the *apn-1*,**

***nth-1* and *ung-1* genes, but not the *exo-3* gene**. The animals were treated for 5 hours with 5-hmU (1 µM) and *apn-1* mRNA assessed by qRT-PCR. **C**, **The expression pattern of the indicated genes during various developmental stages of *C. elegans*.** The data were obtained from the Wormbase.

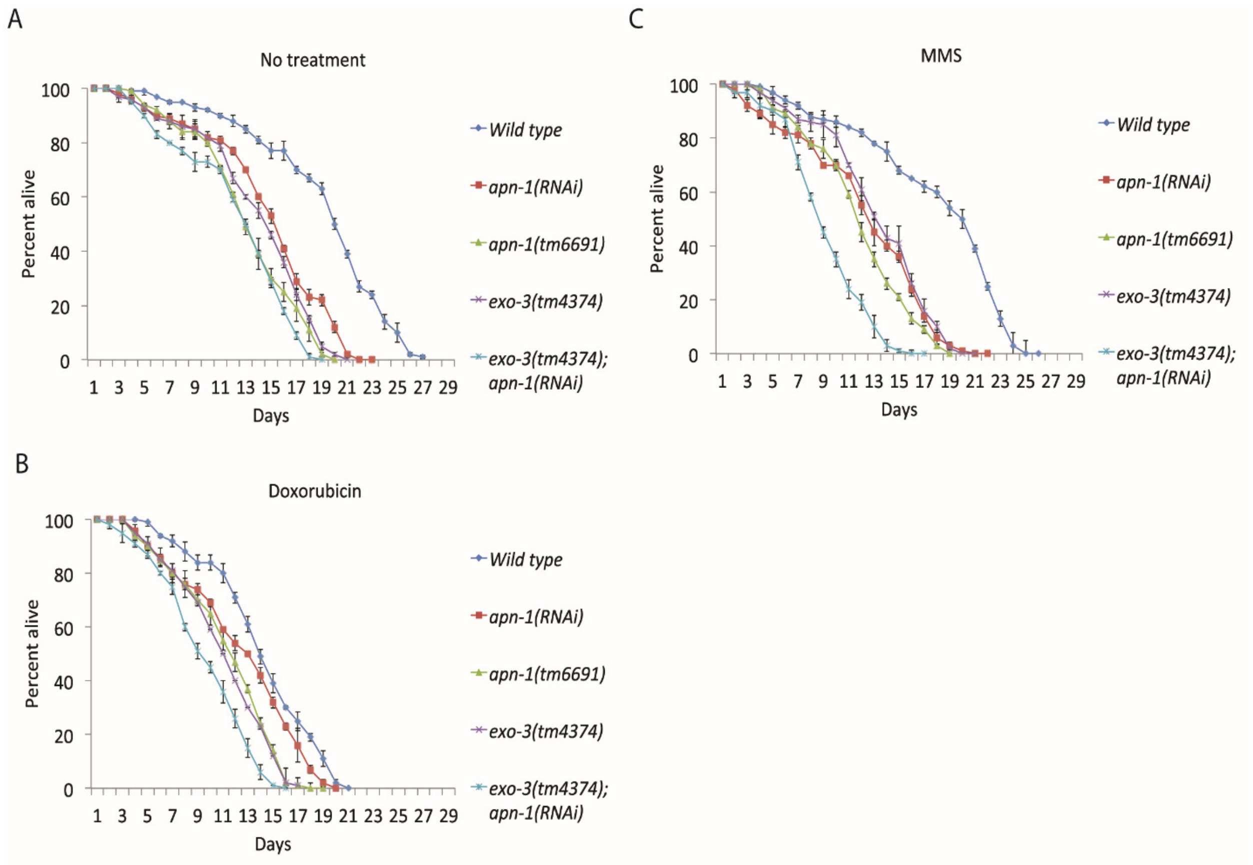

Figure S3. Kaplan-Meier survival plot of BER components exposed to DNA damaging agents. A, B and C, Kaplan-Meier survival plot showing alive percentage of the indicated genotypes. L1-staged animals (*n*=100) were exposed to **A**, No treatment, **B**, DOX and **C**, MMS. Lifespan was blindly assessed starting from young adult animals. The mean lifespan of two independent experiments is shown.

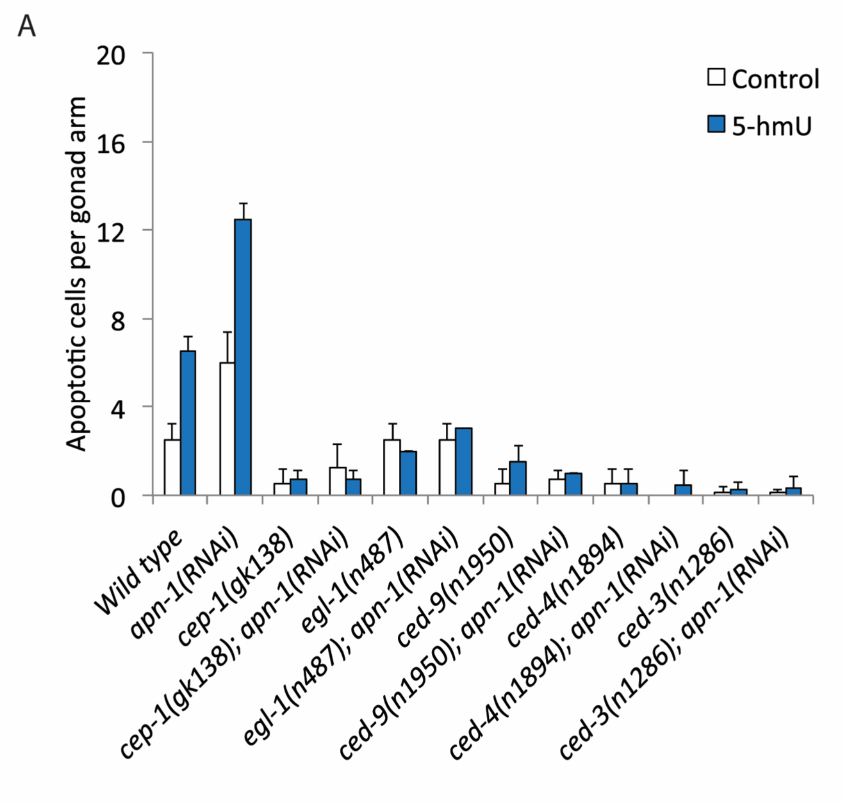

Figure S4. Genetic analysis of control and 5-hmU-induced apoptotic germ cell death. A, Representative chart showing the average quantification of germ cell death in three independent experiments (*n*=30) corresponding to the wild type and the apoptotic defective mutants *cep-1*, *egl-1*, *ced-9*, *ced-4* and *ced-3* without and with RNAi-driven downregulation of *apn-1* in the absence and presence of 5-hmU (1 μM). Apoptotic cell corpses were observed and scored as described in the experimental procedures.

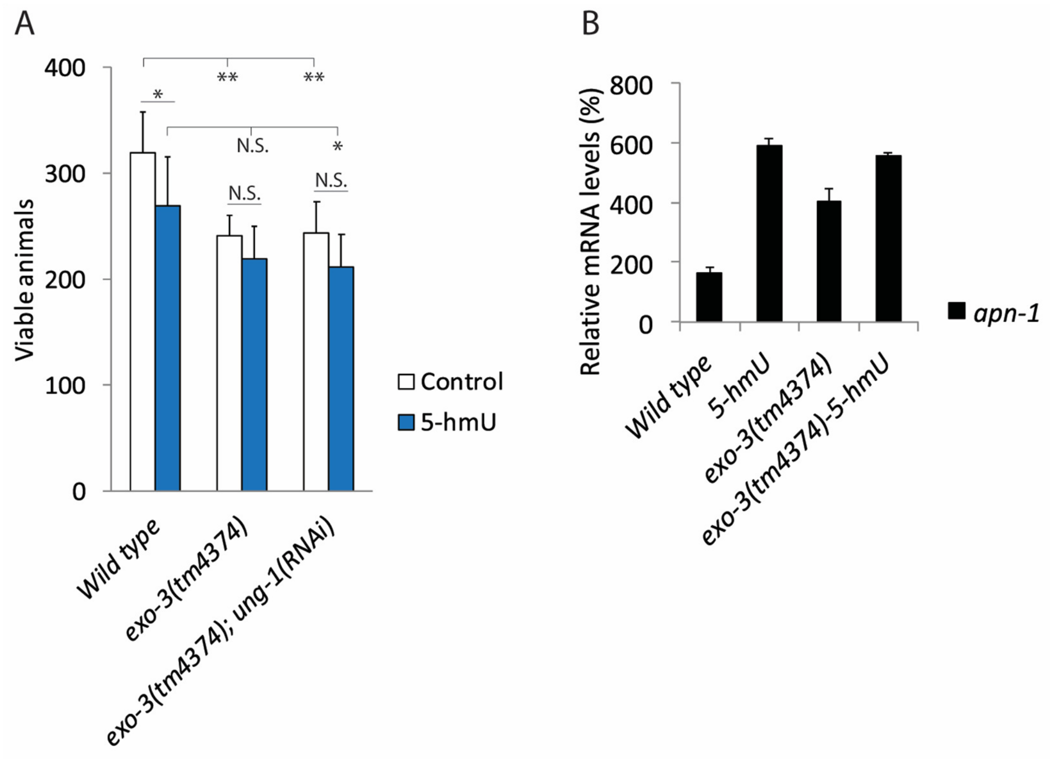

Figure S5. A, *exo-3(tm4374)* mutants are not sensitized to 5-hmU upon ung-1 downregulation by RNAi. Brood size analyses of the indicated genotypes. The data are the mean±S.D. of three independent experiments (*n*=10 monitored for 3 days). Control, Wild type; *exo-3(tm4374)*; and *exo-3(tm4374); ung-1(RNAi)*. **B, *apn-1* expression level in the wild type and *exo-3(tm4374)* mutant without and with 5-hmU exposure.** The animals were untreated or treated for 5 hours with 5-hmU (1 μM) and *apn-1* mRNA assessed by qRT-PCR.

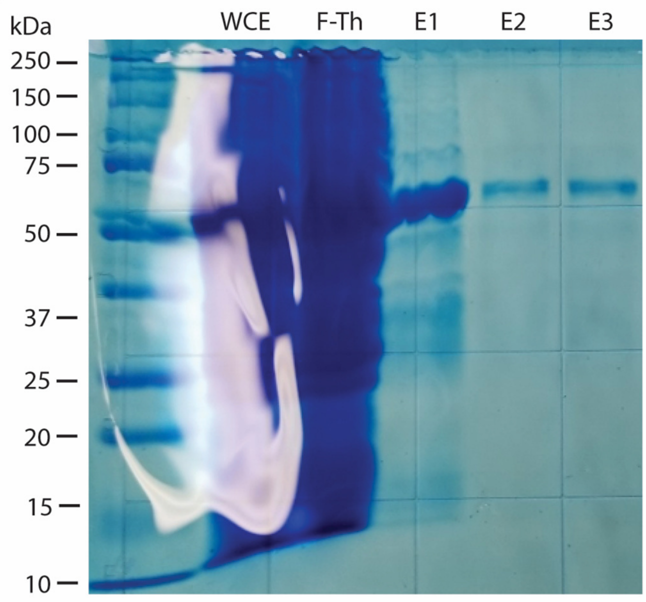

Figure S6. Recombinant GST-UNG-1 purified from an *E. coli* expression system. BL21(DE3) expressing *C. elegans* UNG-1 as a GST-UNG-1 fusion protein was purified according to Nakamura et al., 2008, but using GST-magnetic beads. WCE, whole cell extract, F-Th, flow through from the GST-magnetic beads, E1, E2 and E3 are the elution fractions.

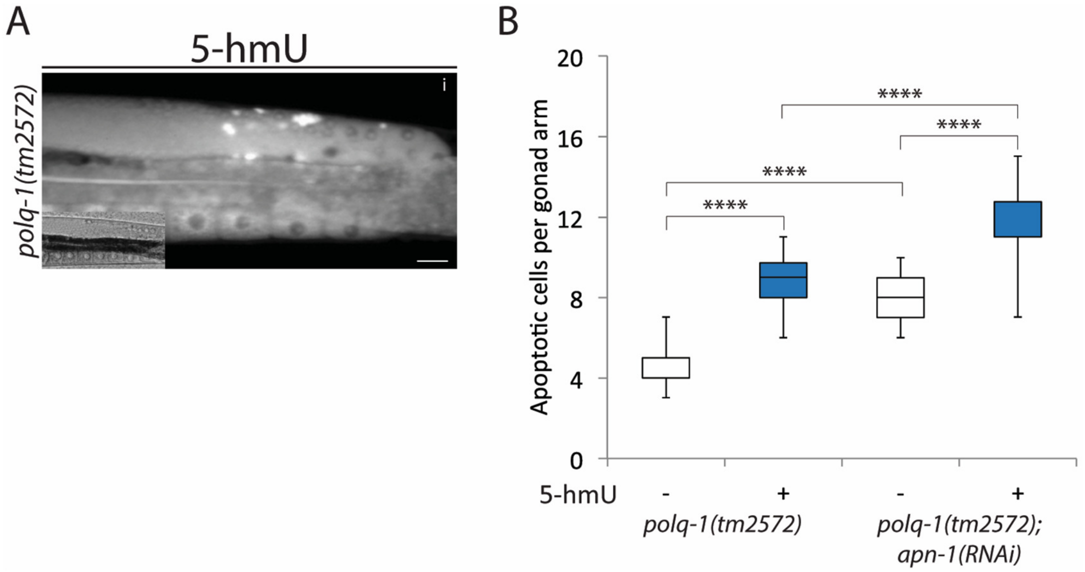

Figure S7. POLQ-1 is required for DNA synthesis after removal of 5-hmU lesions. **A**, Representative images of the posterior gonad arms of the *polq-1* mutant stained with acridine orange upon treatment with 5-hmU as described in Figure 2. **B**, Box and whisker plots showing quantification of germ cell apoptosis in *polq-1(tm2572)* and *polq-1(tm2572); apn-1*(*RNAi*) in the absence and presence of 5-hmU.

```
ceUNG-1      1 MSKTVRIPDMFLKASAASKRKSAS------------------NTENIP-EKVPAGNENQE
hUNG1        1 MIGQKTLYSFFS-PSPARKRHAPSPEPAVQGTGVAGVPEESGDAAAIPAKKAPAGQEEPG
hSMUG1       1 MPQAFLLGSIHE-PAGALMEPQPC-----------------------P------------
consensus    1 M     l smf  psaA krk ps                      iP  k pagne

ceUNG-1     42 VKKMKL---------QAPEPTEIL-----LKSLLTGESWSKLLEE------EFKKGYISK
hUNG1       60 TPPSSPLSAEQLDRIQRNKAAALLRLAARNVPVGFGESWKKHLSG------EFGKPYFIK
hSMUG1      25 -----------------------------------GSLAESFLEEELRLNAELSQLQFSE
consensus   61                q      il        l  Gesw k Lee       Ef k yfsk

ceUNG-1     82 IEKFLNSEVNKGKQVFPPPTQIFTTFNLLPFDEISVVIIGQDPYHDDNQAHGLSFSV---
hUNG1      114 LMGFVA-EERKHYTVYPPPHQVFTWTQMCDIKDVKVVILGQDPYHGPNQAHGLCFSV---
hSMUG1      50 PVGIIY-NPVE-Y-AW-EPHRNYV-TRYCQG-PKEVLFLGMNPGPFGMAQTGVPFGEVSM
consensus  121 imgfl  e  k y vfppPhqift tnlc   ei VvilGqdPyh  nqahGl Fsv

ceUNG-1    139 -------QKGVKPPPS---------LKN------------IYKELESDIEGFKRPDHGNL
hUNG1      170 -------QRPVPPPPS---------LEN------------IYKELSTDIEDFVHPGHGDL
hSMUG1     104 VRDWLGIVGPVLTPPQEHPKRPVLGLECPQSEVSGARFWGFFRNLCGQPEVFFH------
consensus  181        qkpV pPPs         Len            iykeL sdiE F hp hg l

ceUNG-1    171 LGWTRQGVFMLNATLTVRAHEANSHAKIGWQTFTDTVIRIISRQSEKPIVFLLWGGFAHK
hUNG1      202 SGWAKQGVLLLNAVLTVRAHQANSHKERGWEQFTDAVVSWLN-QNSNGLVFLLWGSYAQK
hSMUG1     158 --------------------HCFV-------------------HNLCPLLFLAPSGRNLT
consensus  241  gw rqgv mlna ltvrah ansh   gw  ftd vi  i  qn  plvFLlwggfa k

ceUNG-1    231 KEELIDT--------------------------------------------KKHVVIKTA
hUNG1      261 KGSAIDR--------------------------------------------KRHHVLQTA
hSMUG1     179 PAELPAKQREQLLGICDAALCRQVQLLGVRLVVGVGRLAEQRARRALAGLMPEVQVEGLL
consensus  301 kgelidr                                             kkh Vi ta

ceUNG-1    247 HPSPLSA-RKWWGCKCFSKCNTELENSGRNPINWADL
hUNG1      277 HPSPLSVYRGFFGCRHFSKTNELLQKSGKKPIDWKEL
hSMUG1     239 HPSPRNP-QANKGWEAVA--KERLNELGLLPLLLK--
consensus  361 HPSPls  rgwwGck fsk ne Lq sGr Pi wkdl
```

Figure S8. Sequence alignment of the DNA glycosylases CeUNG-1, hUNG1 and hSMUG1 belonging to *C. elegans* and *H. sapiens*, respectively. Numbers indicate amino acid positions. Identical or similar amino acid residues amongst the sequences are shaded in black or gray, respectively. Dashes indicate gaps. The residues in green are unique to CeUNG-1 and hSMUG1, but not hUNG1.
